# CDC6/THBS1 accelerates pancreatic cancer progression via AKT-mediated glycolytic reprogramming

**DOI:** 10.1038/s41419-026-08758-2

**Published:** 2026-04-21

**Authors:** Mengqiu Yin, Xi Chen, Ziyu Liu, Chongyu Wang, Tianyu Chen, Jinhui Zhu

**Affiliations:** 1 Zhejiang University School of Medicine, Hangzhou, China; 2 Department of Hepatobiliary Pancreatic Surgery, The Second Affiliated Hospital, Zhejiang University School of Medicine, Hangzhou, China; 3 Department of Pathology, The Second Affiliated Hospital, Zhejiang University School of Medicine, Hangzhou, China

**Keywords:** Pancreatic cancer, Cancer metabolism

## Abstract

Pancreatic cancer remains one of the most aggressive malignancies, characterized by early metastatic spread and intrinsic resistance to chemotherapy, which ultimately results in poor treatment outcomes. While the Cell Division Cycle 6 (CDC6) protein has been extensively characterized across multiple cancer types, its functional role in the pathogenesis of pancreatic cancer remains poorly understood. In this study, we performed bioinformatics analysis using RNA-seq data from The Cancer Genome Atlas (TCGA) pancreatic adenocarcinoma cohort, and identified differentially expressed genes through microarray profiling. We conducted a comprehensive functional characterization of CDC6 using CCK-8, colony formation, wound healing, Transwell assays, and flow cytometry, and assessed cellular glycolysis levels based on measurements of ATP production, lactic acid generation, and glucose content. Subcutaneous xenograft mouse models were established to evaluate the impact of CDC6 on tumor growth in vivo, while mechanistic investigations were carried out using co-immunoprecipitation, chromatin immunoprecipitation, dual-luciferase reporter assays, and nucleocytoplasmic fractionation. Our results revealed that CDC6 expression is upregulated in pancreatic cancer, and its elevated expression is significantly correlated with unfavorable patient prognosis. Functional experiments demonstrated that CDC6 promotes the proliferation, migration, and invasion of pancreatic cancer cells. Thrombospondin 1 (THBS1) was identified to be positively correlated with CDC6 expression, and differentially expressed genes were notably enriched in the glucose metabolism pathway. Mechanistically, CDC6 cooperates with E2F1 to facilitate the transcription of THBS1, and the AKT signaling pathway is activated via the CDC6/THBS1 interaction. Overexpression of CDC6 significantly promoted glycolysis and tumor progression in pancreatic cancer, whereas these pro-tumor effects were markedly abrogated by THBS1 knockdown. Collectively, our findings demonstrate that CDC6/THBS1/AKT signaling drives glycolysis and accelerates pancreatic cancer progression, suggesting that the CDC6/THBS1/AKT axis may serve as a promising therapeutic target for pancreatic cancer.

## Introduction

Pancreatic cancer is one of the most aggressive and lethal malignancies, characterized by rapid progression and dismal prognosis [1, 2]. Globally representing the 11th most prevalent cancer diagnosis, it disproportionately accounts for nearly 5% of cancer-related mortality, currently ranking as the sixth leading cause of cancer deaths worldwide [3]. This grim outlook stems principally from diagnostic challenges—a majority of patients present with locally advanced or metastatic disease at initial diagnosis due to the organ’s deep anatomical location and insidious symptom onset [4]. Surgical resection with adjuvant chemotherapy remains the cornerstone of curative treatment, yet only 15–20% of newly diagnosed patients meet resectability criteria [5, 6]. Alarmingly, 75% of surgically treated patients develop recurrence within 24 months post-intervention [7]. For patients who are not candidates for surgical intervention, gemcitabine-based chemotherapy remains the standard first-line treatment; however, the development of drug resistance poses a significant challenge [8], and the incremental therapeutic benefits observed with various combination regimens have been modest [9, 10]. Immune checkpoint inhibitors demonstrate limited efficacy in patients with pancreatic cancer, a phenomenon largely attributed to the high degree of tumor heterogeneity and the immunosuppressive nature of the tumor microenvironment [11]. While molecular mechanisms underlying pancreatic cancer progression remain incompletely understood, there is currently a paucity of effective therapeutic targets available for clinical application [12, 13]. Therefore, there is an urgent need to deepen our understanding of the molecular mechanisms driving pancreatic cancer progression. This will aid in developing more effective targeted therapies and improving the overall prognosis for patients.

Cell Division Cycle 6 (CDC6) functions as an essential licensing factor orchestrating DNA replication initiation through helicase loading and replication checkpoint activation, fundamentally regulating cellular proliferation [14]. Emerging translational studies have mechanistically implicated CDC6 in driving oncogenic phenotypes—including enhanced proliferative capacity, metastatic potential, and metabolic adaptation— across diverse malignancies [15, 16]. Systematic multi-omics profiling demonstrates conserved CDC6 upregulation in 17 solid tumor types (TCGA pan-cancer analysis), with strong clinicopathological correlations to advanced TNM staging and reduced disease-free survival [17–21]. Notably, the overexpression of CDC6 genes is associated with alterations in certain cellular signaling pathways, such as the PI3K/AKT pathway and the JAK2/STAT3 signaling cascade, which in turn may expedite the genesis and the progression of malignancy [22]. Although certain studies have highlighted the regulatory role of CDC6 in pancreatic cancer progression [15], the specific functions and underlying mechanisms of CDC6 in this context remain to be elucidated.

Pancreatic adenocarcinoma exhibits marked divergence in glucose metabolism compared to normal tissues, predominantly manifesting as constitutive aerobic glycolysis (Warburg effect) regardless of oxygen availability [23]. This metabolic rewiring not only supports rapid ATP generation but also maintains redox homeostasis, provides biosynthetic precursors, and activates pro-survival signaling cascades—collectively fostering an immunosuppressive tumor microenvironment that accelerates disease progression [24]. Compelling evidence delineates the PI3K/AKT/mTOR axis as a master regulator of oncogenic metabolism, with phosphoproteomic studies demonstrating AKT-mediated phosphorylation of glycolytic enzymes (e.g., HK2 at Thr473) [25–27]. Within pancreatic cancer systems, the AKT signaling pathway activation orchestrates glycolytic reprogramming and enhances cancer cell survival and proliferation, thereby potentiating the tumorigenesis and pathological progression of pancreatic carcinoma [28]. Particularly, CDC6 overexpression induces metabolic reprogramming characterized by upregulated glycolytic flux (Warburg effect) [22].

The objective of the present study was to delineate the biological role of CDC6 in the pathogenesis of pancreatic cancer and to elucidate its downstream mechanisms. Examination of clinical specimens indicated that CDC6 may serve as a marker of tumor progression and a predictor of unfavorable prognosis in patients with pancreatic cancer. In vivo and in vitro experiments underscored the regulatory influence of CDC6 on the progression of pancreatic cancer, exerting effects on both cellular phenotype and glycolytic activity. THBS1 was identified as a potential downstream target of CDC6, mediating the AKT pathway to regulate glycolysis and influence the development of pancreatic cancer. In summary, this study corroborates the regulatory function of the CDC6/THBS1 axis in modulating glycolysis and tumor progression in pancreatic cancer, thereby highlighting the CDC6/THBS1/AKT axis as a promising therapeutic target in the treatment of this disease (Supplementary Fig. S).

## Materials and methods

### Human tissues

The human pancreatic cancer tissues and paired paracancerous tissues (*n* = 10 patients) used in this study were obtained from the Department of Hepatobiliary and pancreatic surgery, Second Affiliated Hospital of Zhejiang University School of Medicine, Zhejiang, China. This batch of samples was designed to preliminarily assess the differential expression of CDC6 in tumor and adjacent non-tumor tissues. The sample size was constrained by the limited availability of high-quality, paired tumor and adjacent tissue specimens with complete clinical data among pancreatic cancer cases accessible to our research group between May and December 2023. Ultimately, a total of 10 consecutive cases meeting the inclusion criteria were enrolled in the study.

The tissue microarrays (HPanA170Su04-M-009, *n* = 165; HPanA180Su03, *n* = 170) used in this study were purchased from Shanghai Outdo Biotech Co., Ltd. (http://www.superchip.com.cn/index.html). Written informed consent of all individuals has been obtained.

### Immunohistochemistry

Paraffin-embedded tissue blocks were sectioned at 3-μm thickness onto poly-L-lysine-coated slides. Following sequential processing through xylene deparaffinization and ethanol gradient rehydration, heat-induced epitope retrieval was performed using sodium citrate buffer (pH 6.0) with microwave irradiation. Endogenous peroxidase activity was quenched by 3% hydrogen peroxide/methanol solution.

After blocking with 5% species-matched serum (15 min), slides were incubated overnight at 4 °C with anti-CDC6 primary antibody (Doctoral Biotech, 1:200). Signal detection was achieved using biotinylated secondary antibody (Doctoral Biotech, 1:500, 1 h RT) followed by DAB chromogenic visualization. Counterstaining with Harris hematoxylin preceded permanent mounting with resinous medium.

Immunoreactivity was quantified via histoscore (H-score) methodology: H-score = Σ (intensity score × proportion score)

Intensity stratification: 0 (negative), 1 (weak), 2 (moderate), 3 (strong)

Proportion categories: 0 (0%), 1 (1–25%), 2 (26–50%), 3 (51–75%), 4 (76–100%)

Dual independent evaluation by board-certified pathologists in a blinded manner ensured scoring reproducibility (Cohen’s κ = 0.86).

### RNA sequence data processing and screening of differentially expressed genes

To elucidate molecular drivers of pancreatic carcinogenesis and progression, we performed a multi-faceted investigation integrating transcriptomic profiling with clinical outcome analysis.

For transcriptomic characterization, paired pancreatic ductal adenocarcinoma (PDAC) and normal tissue samples were retrieved from The Cancer Genome Atlas (TCGA) through the Genomic Data Commons portal (https://portal.gdc.cancer.gov/, accessed October 10, 2022). Raw RNA-seq counts were normalized and subjected to differential expression analysis using DESeq2 (v1.38.3) in R, with significance thresholds set at |log2 fold change | > 1 and adjusted *p*-value < 0.05.

Parallel prognostic evaluation utilized clinically annotated PDAC samples from cBioPortal (https://www.cbioportal.org/, accessed October 10, 2022). After data harmonization of clinical records, RSEM-normalized expression values were log2(x + 1)-transformed. CDC6 expression levels were dichotomized using a cohort median cutoff, generating high- and low-expression subgroups. Survival disparities were assessed via Kaplan-Meier analysis with log-rank testing, evaluating both overall survival (OS) and progression-free survival (PFS) endpoints.

### Searching for transcription factors

To delineate transcriptional regulation of THBS1, we retrieved its 2-kb upstream regulatory region from the UCSC Genome Browser (https://genome-asia.ucsc.edu/) as putative cis-regulatory elements for in silico prediction of transcription factor binding sites. TFBS prediction was performed using AnimalTFDB 3.0 (http://bioinfo.life.hust.edu.cn/AnimalTFDB/#!/tfbs_predict) with stringent parameters. Complementarily, protein-protein interaction network analysis was conducted via STRING (v11.5; https://string-db.org/) to identify putative functional associations between candidate transcription factors and the upstream gene CDC6, applying a high-confidence interaction score threshold.

### Whole-genome expression microarray

RNA isolation was performed using TRIzol reagent (Invitrogen) in isogenic PATU8988 cell models: shCtrl (lentiviral negative control) and shCDC6 (Knockdown of CDC6). RNA integrity was verified through NanoDrop quantification (A260/A280: 1.7-2.2), followed by two-round cDNA synthesis using SuperScript IV Reverse Transcriptase (Thermo Fisher). Double-stranded cDNA templates were subjected to in vitro transcription with biotinylated ribonucleotides via a T7 promoter-based IVT kit (Thermo Fisher), generating labeled cRNA. Following purification with RNeasy columns (Qiagen), fragmented cRNA probes (35-200 bp) were hybridized to GeneChip Human Genome U133 Plus 2.0 arrays (Affymetrix) under standardized conditions (45 °C, 16 h rotation). Post-hybridization processing, including washing, staining and array scanning, was automated through GeneChip Scanner 3000 7G (Affymetrix) following the manufacturer’s protocols.

The microarray data were preprocessed and normalized using the Robust Multi-array Average (RMA) algorithm. Differential gene expression analysis was subsequently performed using the limma package (version 3.52.4) in the R environment. Differentially expressed genes (DEGs) were identified using threshold criteria of adjusted *p*-value < 0.05 and absolute fold change ≥2.

For functional annotation, we conducted comprehensive enrichment analyses through clusterProfiler (version 4.7.1.1), interrogating both Gene Ontology (GO) biological processes and Kyoto Encyclopedia of Genes and Genomes (KEGG) pathways. Complementarily, gene set enrichment analysis (GSEA) was performed using the GSEA software (Linux version 4.2.3) with the C2 curated gene sets collection from the MSigDB database (downloaded January 2023). Significance thresholds were established at a false discovery rate (FDR) < 0.25 for pathway enrichment.

All experimental procedures were executed with three independent biological replicates. Statistical comparisons between groups were performed using a two-tailed Student’s *t* test, with *p*-values < 0.05 considered statistically significant. Data visualization and statistical computations were implemented in R (version 4.2.2).

### Cell culture

The human pancreatic cancer cell lines PATU8988, SW1990, PANC-1, BxPc-3, QGP-1, along with 293 T embryonic kidney cells and HPDE6-C7 normal pancreatic ductal epithelial cells, were obtained from the Shanghai Cell Bank of Chinese Academy of Sciences. These cell lines were authenticated via short tandem repeat (STR) profiling prior to purchase, and mycoplasma contamination was excluded through routine testing. Cell culture maintenance was performed according to medium-specific requirements: SW1990 in Minimum Essential Medium (MEM); PATU8988, 293 T, and PANC-1 in Dulbecco’s Modified Eagle Medium (DMEM); BxPc-3, QGP-1, and HPDE6-C7 in Roswell Park Memorial Institute (RPMI) 1640 medium (all media from Gibco, Thermo Fisher Scientific).

All culture media were supplemented with 10% (v/v) heat-inactivated fetal bovine serum (FBS) and 1% penicillin-streptomycin antibiotic mixture (100 U/ml penicillin, 100 μg/ml streptomycin). Cells were maintained in a humidified 5% CO2 atmosphere at 37 °C, with medium refreshment every 48–72 h based on confluence status.

### Determination of sample size for cell culture experiments

The sample size, defined as the number of independent repeated experiments, was determined in accordance with established practices within the relevant research field and informed by the variability observed in preliminary experiments conducted in our laboratory. This approach ensured sufficient sensitivity to detect consistent trends and statistically significant differences. All key experiments were performed with a minimum of three independent replicates.

### Polymerase chain reaction (PCR)

Total RNA was isolated from cultured cells using TRIzol™ reagent (Sigma-Aldrich) following the manufacturer’s protocol. RNA purity and concentration were quantified using a NanoDrop 2000 spectrophotometer (Thermo Fisher Scientific), with acceptable samples demonstrating 260/280 nm absorbance ratios >1.8.

For cDNA synthesis, 2 μg of total RNA was subjected to genomic DNA elimination and reverse transcription using the HiScript® Q RT SuperMix ( + gDNA wiper) kit (Vazyme Biotech) according to the manufacturer’s instructions.

Quantitative real-time PCR amplification was performed in triplicate using AceQ® qPCR SYBR Green Master Mix (Vazyme Biotech) on a QuantStudio 7 Pro Real-Time PCR System (Applied Biosystems). Thermal cycling conditions consisted of: 95 °C for 5 min; 40 cycles of 95 °C for 10 sec and 60 °C for 30 s; followed by melt curve analysis. GAPDH served as the endogenous control for normalization, with primer sequences detailed in Table 1S.

### Western blot

Cellular proteins were extracted using ice-cold RIPA lysis buffer (Beyotime) supplemented with 1× protease inhibitor cocktail. Protein concentrations were quantified using the BCA Protein Assay Kit (Beyotime) according to the manufacturer’s protocol.

Equal protein aliquots (20 μg per lane) were resolved by sodium dodecyl sulfate-polyacrylamide gel electrophoresis (SDS-PAGE) using 10–15% separating gels with 5% stacking gels, followed by electrophoretic transfer to polyvinylidene difluoride (PVDF) membranes (Millipore). Membranes were blocked with 5% (w/v) non-fat dry milk in Tris-buffered saline containing 0.1% Tween-20 (TBST) for 1 h at room temperature.

Primary antibody incubations were performed overnight at 4 °C with gentle agitation, followed by three 5-min TBST washes. Horseradish peroxidase (HRP)-conjugated secondary antibodies were applied for 1 h at room temperature. Antibody specifications are detailed in Supplementary Table 2S.

Protein signals were developed using the Enhanced Chemiluminescence (ECL) HRP Substrate Kit (Beyotime) and captured using the Amersham Imager 600 system (GE Healthcare). Band intensities were quantified using ImageJ software (version 1.53t, NIH) normalized to β-actin or GAPDH or Histone H3 loading controls.

### Construction, packaging, and transfection of lentiviral plasmids

Lentiviral constructs, including scramble shRNA control (shCtrl/NC), CDC6-targeting shRNA (shCDC6), THBS1-targeting shRNA (shTHBS1), and CDC6 overexpression vectors, were obtained from Yibei Rui Biotechnology Co., Ltd (Shanghai, China). For knockdown experiments, shRNA sequences were cloned into pLVX-EGFP lentiviral vectors (Clontech) using AgeI/EcoRI restriction sites. The CDC6 coding sequence (NCBI accession: NM_001254) and THBS1 coding sequence (NCBI accession: NM_003246.2)were synthesized and subcloned into pLVX-Puro overexpression vectors (Takara Bio) for ectopic expression.

Viral transduction was performed by seeding target cells in 6-well plates at 1 × 10^5 cells/well and culturing under standard conditions (37 °C, 5% CO2). When reaching 30-50% confluency, cells were infected with lentiviral particles at a multiplicity of infection (MOI) of 10 in complete medium. After 24 h incubation, viral supernatants were replaced with fresh complete medium. Transduction efficiency was verified 48 h post-infection by EGFP fluorescence microscopy (Olympus IX73).

Stable cell lines were selected using 2 μg/ml puromycin (Sigma-Aldrich) treatment for 72 h, with selection maintained throughout subsequent experiments. All shRNA target sequences are provided in Table 3S.

### Dual luciferase reporter gene assay

Bioinformatic analysis using the Human Transcription Factor Database (http://bioinfo.life.hust.edu.cn/HumanTFDB) predicted two putative E2F1 binding motifs within the THBS1 promoter: 5’-CGCCGAGCCCGTGCGCGCCAAG-3’ (Site 1) and 5’-GCGCGCCAGG-3’ (Site 2). Site-directed mutagenesis was performed to generate THBS1 promoter mutants (Site 1: 5’-TGCCGAGCCCGTATATATTGGA-3’; Site 2: 5’-ATATATTGAA-3’) for functional validation.

HEK293T cells were seeded in 96-well plates at 5×10³ cells/well and cultured to 50-70% confluency. Cells were co-transfected with 0.2 μg/well of either wild-type (WT) or mutant (MUT) THBS1 promoter-luciferase reporter plasmids (pGL4.10, Promega), in combination with pcDNA3.1-E2F1 overexpression vector or empty vector controls (Addgene #12351) using Lipofectamine 3000 reagent (Thermo Fisher Scientific). Following 48 h incubation at 37 °C/5% CO₂, dual-luciferase activity was quantified using the Dual-Luciferase® Reporter Assay System (Promega, E1910) according to manufacturer specifications. Firefly luciferase signals were normalized to co-transfected Renilla luciferase activity (phRL-TK vector, Promega) using a SpectraMax M5 microplate reader (Molecular Devices). Relative promoter activity was calculated as the firefly/Renilla luminescence ratio.

### Determination of glycolysis levels

Cellular glucose uptake was quantified using the Glucose Assay Kit (Solarbio, BC2500) following enzymatic colorimetric methods. Intracellular ATP levels were measured with the ATP Content Assay Kit (Solarbio, BC0300) based on luciferase bioluminescence. Lactate production was determined using the Lactate Assay Kit (Solarbio, BC2230) via enzymatic cycling reactions. For all metabolic assays, cells were lysed using ice-cold RIPA buffer and processed according to the manufacturer's protocols. Absorbance readings were acquired using a SpectraMax M5 microplate reader (Molecular Devices) with specific wavelengths: 505 nm (glucose), 636 nm (ATP), and 570 nm (lactate). Extracellular acidification rate (ECAR) was monitored in real-time using the pH-sensitive Fluorescent Probe Assay Kit (Solarbio, SM8010). Briefly, 8 × 10^4^ cells/well were seeded in 96-well black-walled plates and loaded with probe according to kit instructions. Fluorescence intensity (Ex/Em = 540/590 nm) was recorded every 4 min for 60 min using a Synergy H1 hybrid reader (BioTek).

Glycolysis-related proteins (FBP1, G6PD, GLUT1, HK2, PKM2) were analyzed by western blot as previously described, with GAPDH serving as loading control.

### CCK-8 assay

Cell proliferation was assessed using the Cell Counting Kit-8 (CCK-8, Sigma-Aldrich, 96992) according to optimized protocols. Cells were seeded in 96-well plates at 2.5 × 10³ cells/well (100 μL complete medium/well) and cultured under standard conditions (37 °C, 5% CO₂). At designated time points (24, 48, 72, 96, and 120 h), 10 μL CCK-8 reagent was added to each well, followed by 2 h incubation protected from light. The water-soluble formazan product generated by cellular dehydrogenases was quantified by measuring absorbance at 450 nm using a Tecan Infinite M200 PRO microplate reader. Three technical replicates were performed per condition, with blank medium controls included in each plate. Proliferation curves were generated by normalizing absorbance values to day 0 baseline measurements.

### Wound-healing assay

Cells were seeded in 96-well plates at 5 × 10⁴ cells/well in complete growth medium and cultured to 90% confluence. Uniform linear scratches were generated using a sterile 200-μl pipette tip, followed by two gentle washes with Phosphate Buffered Saline (PBS) to remove dislodged cells. To suppress proliferation and isolate migration effects, cells were maintained in serum-free medium under standard culture conditions (37 °C, 5% CO₂). Initial reference images were acquired using a Thermo Scientific Cellomics CX7 High-Content Scanner equipped with a 10× objective. After 12 or 24 h incubation, the same fields were reimaged under identical settings. Scratch widths were quantified using ImageJ software (NIH) with the MRI Wound Healing Tool plugin.

Migration rate (%) = [(W₀ − W₂₄)/W₀] × 100 Where: W₀ = Mean scratch width at 0 h W₂₄ = Mean scratch width at 24 h

### Transwell assay

Cell migration capacities were assessed using 24-well Transwell® chambers (Corning, 8 μm pore size, CLS3422). The inserts were rehydrated with 200 μL serum-free medium for 30 min prior to cell seeding. Cells were serum-starved in 0.5% FBS medium for 12 h before trypsinization. After PBS washing, 1 × 10⁵ cells in 100 μL serum-free medium were seeded in the upper chamber, while 600 μL complete medium containing 10% FBS was added to the lower compartment as a chemoattractant. Following 24 h incubation at 37 °C/5% CO₂, non-migrated cells on the upper membrane surface were mechanically removed using cotton-tipped swabs.

Migrated cells were fixed with 4% paraformaldehyde (RT, 15 min) and stained with 0.1% crystal violet (RT, 15 min). Five random 200× fields per insert were imaged using an Olympus IX73 inverted microscope equipped with a DP80 CCD. Quantitative analysis was performed using ImageJ software with the Cell Counter plugin.

### Clone formation assay

Cells in logarithmic growth phase were seeded into 6-well plates at a density of 500 cells/well in 2 mL complete growth medium per well. Plates were maintained under standard culture conditions (37 °C, 5% CO₂) for 14 days with medium replacement every 2–3 days. Following incubation, colonies were washed twice with sterile water and fixed with 4% (w/v) paraformaldehyde (China National Pharmaceutical Group Chemical Reagent Co., Ltd.) for 15 min at room temperature. Fixed colonies were stained with 0.1% (w/v) crystal violet solution (Dingguo Biotechnology, Shanghai, China) for 30 min, followed by three sterile water washes to remove excess dye. Air-dried plates were scanned using an EPSON Perfection V800 flatbed scanner at 1200 dpi resolution. Colonies containing ≥50 cells were quantified using ImageJ software with the ColonyArea plugin. Three biological replicates were performed per experimental condition.

### Cell apoptosis assay

Cell apoptosis was assessed using the eBioscience™ Annexin V-APC Apoptosis Detection Kit (Thermo Fisher Scientific). Cells were cultured in 6-well plates until reaching 85% confluence, at which point apoptosis was induced through drug treatment. Both adherent and floating cell populations were harvested by combining supernatant-derived cells with trypsinized adherent cells. The cell suspension was centrifuged at 300 × *g* (1500 rpm) for 5 min at 4 °C, followed by sequential washing with ice-cold PBS and Annexin-binding buffer. Washed cells were resuspended in staining buffer to achieve a final concentration of 1 × 10⁶ cells/mL. Subsequently, 100 μL aliquots were stained with 5 μL Annexin V-APC and 5 μL propidium iodide (PI) for 15 min in the dark at room temperature. Following incubation, samples were diluted to 300 μL with fresh staining buffer and analyzed within 1 h using an Attune™ NxT Flow Cytometer (Thermo Fisher Scientific). Initial gating excluded cellular debris based on forward scatter (FSC) vs. side scatter (SSC) characteristics, with fluorescence compensation established using single-stained controls. Apoptotic cell populations (Annexin V + /PI- for early apoptosis; Annexin V + /PI+ for late apoptosis) were quantified as a percentage of total live cells.

### Co-immunoprecipitation (co-IP)

Co-immunoprecipitation (Co-IP) assays were performed to investigate protein-protein interactions in pancreatic cancer cell lines PATU8988 and SW1990. Cells were lysed under standard culture conditions using ice-cold RIPA buffer (Beyotime) supplemented with 1× protease inhibitor cocktail and 1× phosphatase inhibitor cocktail. Lysates were precleared with Protein A/G Plus Agarose beads (Santa Cruz Biotechnology) for 1 h at 4 °C to reduce nonspecific binding. Supernatants were incubated overnight at 4 °C with rotation using 2 μg of target-specific primary antibodies or matched isotype controls. Immune complexes were captured by adding 20 μL Protein A/G Plus Agarose beads and incubating for 4 h at 4 °C with gentle agitation. Beads were pelleted by centrifugation (1000 × *g*, 5 min) and washed four times with lysis buffer to remove unbound proteins. Bound proteins were eluted by boiling in 1 × SDS buffer at 95 °C for 10 min. Eluates were analyzed by Western blot as previously described.

### Chromatin immunoprecipitation (ChIP)

Chromatin immunoprecipitation was performed using the Enzymatic Chromatin IP Kit (#9005, Cell Signaling Technology) following the manufacturer’s guidelines with critical modifications. Cells were fixed with 1% formaldehyde for 10 min at 37 °C, quenched with 0.125 M glycine. Cells were lysed in SDS lysis buffer containing protease inhibitors. Chromatin was digested with 0.5 U/μL micrococcal nuclease (MNase) at 37 °C for 15 min. After termination with 10 mM EDTA, lysates were sonicated (3 cycles of 15 sec pulses at 20% amplitude, Branson Sonifier) to solubilize chromatin, followed by centrifugation (16,000 × *g*, 10 min) to collect soluble chromatin fragments (150-900 bp). Immunoprecipitation was performed overnight at 4 °C with rotation using 5 μg of target-specific antibody or normal rabbit IgG (negative control). Antibody details in Table 4S. Antibody-chromatin complexes were captured with Protein G Magnetic Beads (included in kit), washed sequentially with Low Salt Immune Complex Wash Buffer and High Salt Immune Complex Wash Buffer, then eluted in ChIP Elution Buffer. Input DNA (10% of starting chromatin) and immunoprecipitated DNA were reverse-crosslinked at 65 °C overnight, purified using spin columns, and analyzed by qPCR using primers targeting conserved genomic regions. Enrichment values were normalized to input DNA and expressed as fold change relative to IgG controls.

### Animal experimentation

The sample size (6 mice/group) was determined from relevant literature and pilot variability data, adhering to the “reduction” principle to minimize animal use while ensuring detection of statistically significant, biologically relevant differences.

Female NOD-Prkdcem26Cd52Il2rgem26Cd22/NjuCrl (NCG) mice (6-week-old, *n* = 24) from Jiangsu Jicui Yaokang Biotechnology Co. (SCXK 2022-0012) were maintained in specific pathogen-free (SPF) conditions with 12/12 h light-dark cycles. Following 1-week acclimation, mice were randomly allocated into four experimental groups (*n* = 6/group) using stratified randomization based on body weight: NC (negative control); shTHBS1 (THBS1 knockdown); CDC6-OE (CDC6 overexpression); shTHBS1 + CDC6-OE (combinatorial modulation).

For a supplementary experiment, female BALB/cAnPt-Foxn1^nu^/Foxn1^nu^ (BALB/c Nude) mice (5-week-old, *n* = 24) from Jiangsu Jicui Yaokang Biotechnology Co. (SCXK 2022-0012) were maintained in specific pathogen-free (SPF) conditions with 12/12 h light-dark cycles. Following a 1-week acclimation, mice were randomly allocated into four experimental groups (*n* = 6/group) using stratified randomization based on body weight: NC; CDC6-OE group; CDC6-OE with MK-2206 intraperitoneal injection (CDC6-OE + MK-2206 group); CDC6-OE with 2-DG intraperitoneal injection (CDC6-OE + 2-DG group).

PATU-8988 cells (1 × 10⁷ cells/200 μL) were subcutaneously injected into the right flank using 25 G needles. Post-implantation monitoring included: Daily clinical observation for distress signs; Weekly body weight measurements ( ± 0.1 g). Tumor volume quantification via digital caliper (Mitutoyo) using ellipsoid formula:

$${\rm{V}}={\rm{\pi }}/6\times {\rm{L}}\times {{\rm{W}}}^{2}$$

Where L=long-axis, W=short-axis

At day 14 post-implantation, or day 25 post-implantation for the supplementary experiment, mice were euthanized via CO₂ asphyxiation (30% chamber fill rate) followed by cervical dislocation. Excised tumors were weighed and stored at –80 °C for subsequent experiments.

### Exclusion criteria

Technical failures (RNA < 20 ng/μL, A260/A280 1.8–2.2, RIN < 7, protein <0.5 μg/μL, or non-specific staining/tissue detachment); protocol deviations (anesthesia-related deaths, non-intervention infections, or critical data loss); statistical outliers ( ± 3 SD from group mean); and humane endpoints (weight loss >20%, severe pain, or behavioral abnormalities). All exclusion criteria were prospectively established during the study design phase to ensure methodological rigor and data integrity.

### Method of randomization

This study did not utilize a random assignment approach; instead, samples and experimental animals were allocated to groups based on predetermined treatment protocols.

### Blinding procedure

Unique identification number assigned by an independent operator, concealed group allocation. Treatments (drug administration, transfection) were administered per coded numbers, with agents/controls visually identical. All assessments (histopathology, biochemical, imaging) and data entry were conducted by blinded investigators. The blinding code remained sealed until the final statistical analysis.

### Statistical analysis

All analyses were performed using three complementary platforms: SPSS Statistics (v22.0, IBM), R programming language (v4.0.0, R Foundation), and GraphPad Prism (v9.2.0). Analytical approaches were selected based on data characteristics and distributional assumptions. For continuous variables, Parametric: Student’s *t* test (two-group)/One-way ANOVA with Tukey’s post-hoc (multi-group), Non-parametric: Mann-Whitney U (two-group)/Kruskal-Wallis with Dunn’s correction (multi-group). For categorical variables: Pearson χ² test (expected counts ≥5), Fisher’s exact test (expected counts <5). For correlation analysis, Pearson’s r (linear relationships). For survival analysis, Kaplan–Meier estimator with log-rank Mantel-Cox test. The standard deviation is employed to quantify the variability of data within each group. If the variances are unequal, non-parametric tests should be employed.

Multiple comparison adjustments employed Benjamini-Hochberg FDR control where applicable. All tests were two-sided with α = 0.05 defining statistical significance. Graphical representations were generated using ggplot2 (R) and Prism’s advanced visualization toolkit. *P* value < 0.05 has statistical significance.

## Results

### Expression of CDC6 in pancreatic cancer and its correlation with poor prognosis

**Fig. 1:**
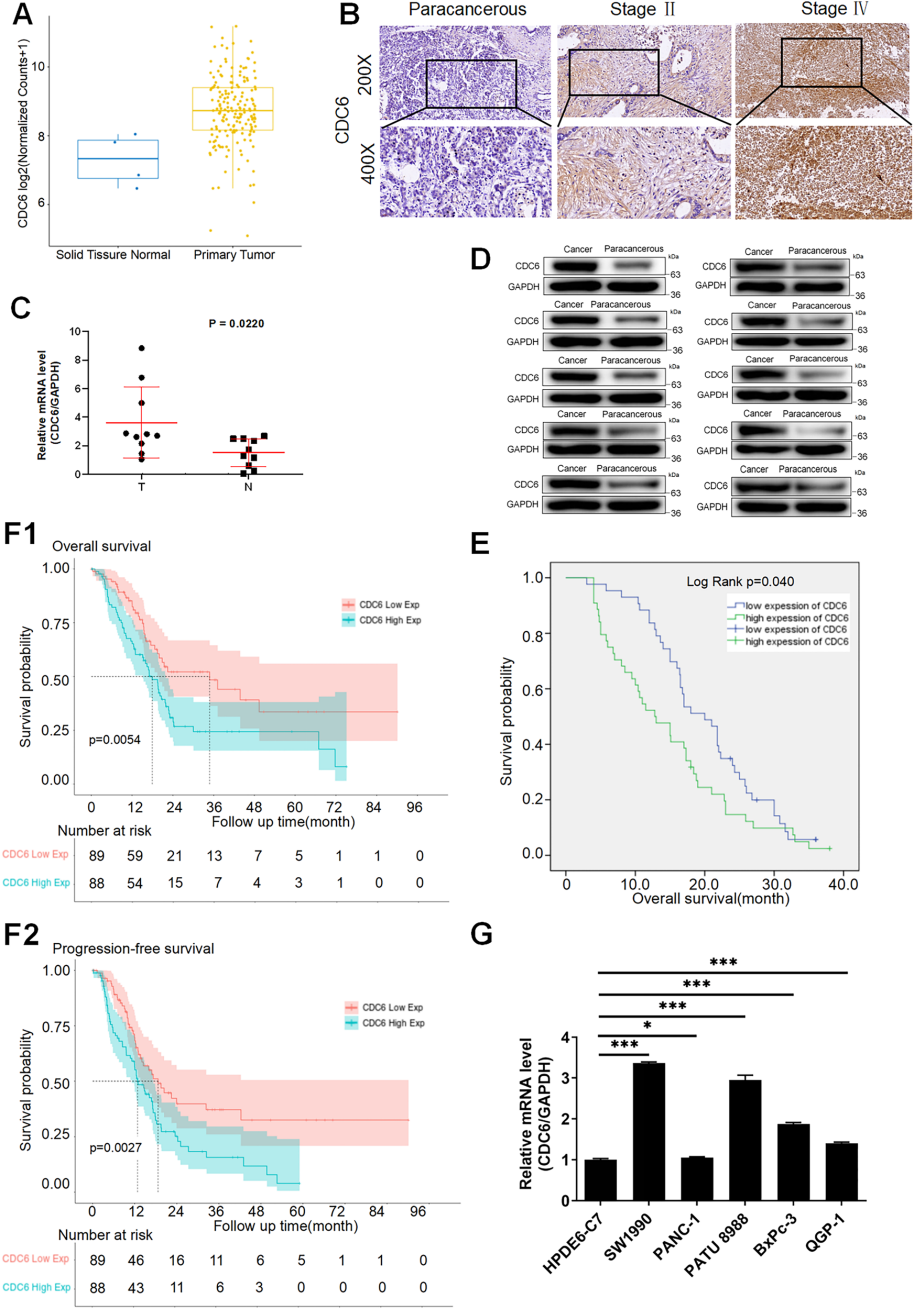
CDC6 overexpression correlates with pancreatic cancer progression and poor prognosis. **A** Upregulation of CDC6 in pancreatic cancer versus normal pancreas tissues from TCGA database. **B** IHC analysis of CDC6 expression in pancreatic cancer tissue microarray containing adjacent tissues and cancer tissues in different stages. **C** qPCR analysis of CDC6 in pancreatic cancer and adjacent tissues (*n* = 10). **D** Protein expression levels of CDC6 in pancreatic cancer and adjacent tissues determined by WB (*n* = 10). **E** Kaplan-Meier curves of survival analysis of patients with different expressions of CDC6 in the tissue microarray (*n* = 165). **F**1–2 Kaplan-Meier curves of survival analysis of patients with different expressions of CDC6 from the TCGA database. **G** Baseline CDC6 expression across pancreatic cancer cell lines, with PATU8988 and SW1990 showing higher overexpression relative to normal ductal cells HPDE6-C7. Data represent mean ± SD.*p < 0.05, ***p < 0.001.

To systematically evaluate CDC6’s clinical relevance in pancreatic cancer, we implemented a multi-platform validation strategy combining bioinformatics analysis with experimental verification. Initial interrogation of TCGA data revealed significant CDC6 upregulation in pancreatic cancer tissues (*P* = 0.0416) (Fig. 1A). Immunohistochemical staining (IHC) was employed to validate the expression levels of CDC6 in pancreatic cancer and adjacent non-tumor tissues using a human tissue microarray with samples from 165 pancreatic cancer patients (HPanA170Su04-M-009). The positive staining intensity of CDC6 in pancreatic cancer tissues was significantly higher compared to that in adjacent non-tumor tissues (*P* < 0.05, Table 1; Fig. 1B). Furthermore, quantitative real-time polymerase chain reaction (qPCR) and Western blot analyses confirmed that both mRNA (*P* = 0.022; Fig. 1C) and protein (*P* < 0.05; Figs. 1D and WB1) levels of CDC6 were upregulated in pancreatic cancer tissues from 10 surgical specimens. Expanding to clinical correlates, Mann-Whitney U and Spearman correlation analyses showed that CDC6 overexpression was correlated with advanced tumor stage and metastatic progression (*P* < 0.001; Tables 2, 3). Furthermore, Kaplan-Meier survival analysis also demonstrated a significant association between elevated CDC6 gene expression and decreased overall survival in the tissue microarray samples (Fig. 1E). Survival analysis indicated that high CDC6 expression was linked to reduced median overall survival (17.92 vs. 34.82 months, HR = 2.31, *P* = 0.00544) and progression-free survival (12.82 vs. 18.67 months, HR = 1.8, *P* = 0.00273) in TCGA data (Fig. 1F). At the cellular level, CDC6 expression was further examined in pancreatic cancer cell lines, with PATU8988 and SW1990 showing higher levels compared to normal HPDE6-C7 cells (Fig. 1G), establishing these for functional studies. Collectively, these findings indicate CDC6’s potential as a prognostic marker and therapeutic target for improving patient outcomes.

**Table 1:** Expression patterns in pancreatic cancer tissues and para-carcinoma tissues were revealed in immunohistochemistry analysis.

CDC6 expression	Tumor tissue		Para-carcinoma tissue		p value
	Cases	Percentage	Cases	Percentage	
Low	43	49.4%	54	69.2%	0.020
High	44	50.6%	24	30.8%

**Table 2:** Relationship between CDC6 expression and tumor characteristics in patients with pancreatic cancer.

Features	Cases	CDC6 expression		p value
		low	high	
All patients	87	43	44	
Age				0.337
≥59years	43	19	24	
<59years	44	24	20	
Gender				0.731
Male	51	26	25	
Female	36	17	19	
Tumor size				0.165
>3.8 cm	44	25	19	
≤3.8 cm	43	18	25	
Stage				<0.001
I	24	17	7	
II	28	20	8	
III	9	4	5	
IV	26	3	24	
T Infiltrate				0.049
T1	6	4	2	
T2	38	22	16	
T3	33	14	19	
T4	10	3	7	
Metastasis				<0.001
M0	63	40	23	
M1	24	3	21

**Table 3:** Relationship between CDC6 expression and tumor characteristics in patients with pancreatic cancer.

	CDC6 expression (*n* = 87)	
	**Pearson correlation**	**Significance (two-tailed)**
Stage	0.509	*P* < 0.001
Metastasis	0.456	*P* < 0.001

### CDC6 depletion suppresses malignant progression in pancreatic cancer

**Fig. 2:**
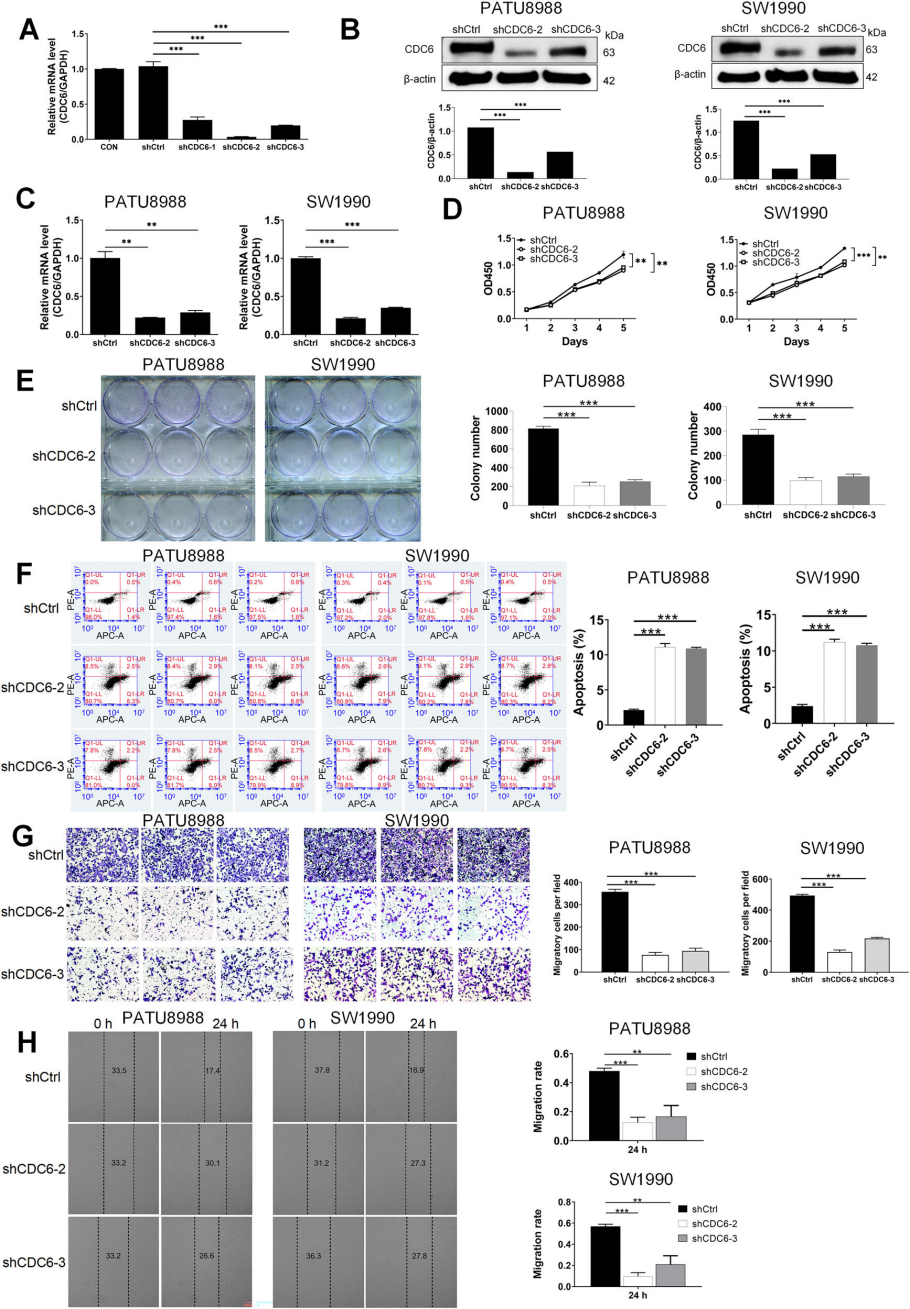
CDC6 knockdown suppresses malignant phenotypes of pancreatic cancer cells in vitro. **A** Validation of CDC6 knockdown efficiency by qPCR. CDC6 was knocked down in PATU8988 and SW1990 cells by lentiviral transfection and validated at (**B**) the protein level by Western blot and **C** mRNA level by qPCR. **D** Cell viability assessed by CCK-8 assay on PATU8988 and SW1990 cells after CDC6 knockdown. **E** Clone formation assay detected the cell reproductive capacity. **F** Apoptosis of PATU8988 and SW1990 cells after knockdown of CDC6 was measured by Annexin V/PI flow cytometry. **G** Migration capacity was assessed by Transwell assay in PATU8988 and SW1990 cells after CDC6 knockdown. **H** Migration potential evaluated by wound healing assay in PATU8988 and SW1990 cells after CDC6 knockdown. **P < 0.01, ***P < 0.001. Data represent mean ± SD.

To investigate the functional role of CDC6 in pancreatic cancer progression, we established stable knockdown models in PATU8988 and SW1990 cells using lentiviral shRNAs. Among the candidates, shCDC6-2 and shCDC6-3 achieved the highest silencing efficiency, reducing the expression of CDC6 mRNA, respectively (*P* < 0.001), and were selected for subsequent experiments (Fig. 2A). Transduction efficiency exceeded 80%, as confirmed by fluorescence microscopy (Supplementary Fig. S1A, B). Subsequently, the result of qPCR and WB validated significant CDC6 depletion at both protein levels (*P* < 0.001 in both PATU8988 and SW1990; Figs. 2B and WB2) and mRNA (*P* < 0.01 in PATU8988; *P* < 0.001 in SW1990; Fig. 2C) compared to shCtrl groups. A series of functional experiments was performed to investigate the impact of CDC6 knockdown on the phenotypic characteristics of pancreatic cancer cells. The Cell Counting Kit-8 (CCK-8) assay exhibited slower proliferation rates in the shCDC6-2 and shCDC6-3 groups compared to controls (*P* < 0.01 in PATU8988; *P* < 0.001 in SW1990; Fig. 2D). Similarly, colony formation was significantly decreased in CDC6-depleted cells (*P* < 0.01 in both cell lines; Fig. 2E). Flow cytometry further demonstrated a notable increase in apoptosis rates in the shCDC6-2 and shCDC6-3 groups compared to controls (*P* < 0.01 in both cell lines; Fig. 2F). Migration capacity was also affected, as evidenced by wound healing assays, which showed a significant reduction in migration rates after 24 h in both knockdown groups (*P* < 0.01 in both cell lines; Fig. 2G). Similarly, Transwell assays showed decreased migration rates of shCDC6-2 and shCDC6-3 cells compared to the shCtrl groups (*P* < 0.001 in both cell lines; Fig. 2H). Collectively, these findings underscore the pivotal role of CDC6 in promoting proliferation and metastatic behavior in pancreatic cancer cells, highlighting its potential as a therapeutic target.

### CDC6 promotes pancreatic cancer progression by upregulating THBS1 expression through interaction with E2F1

**Fig. 3:**
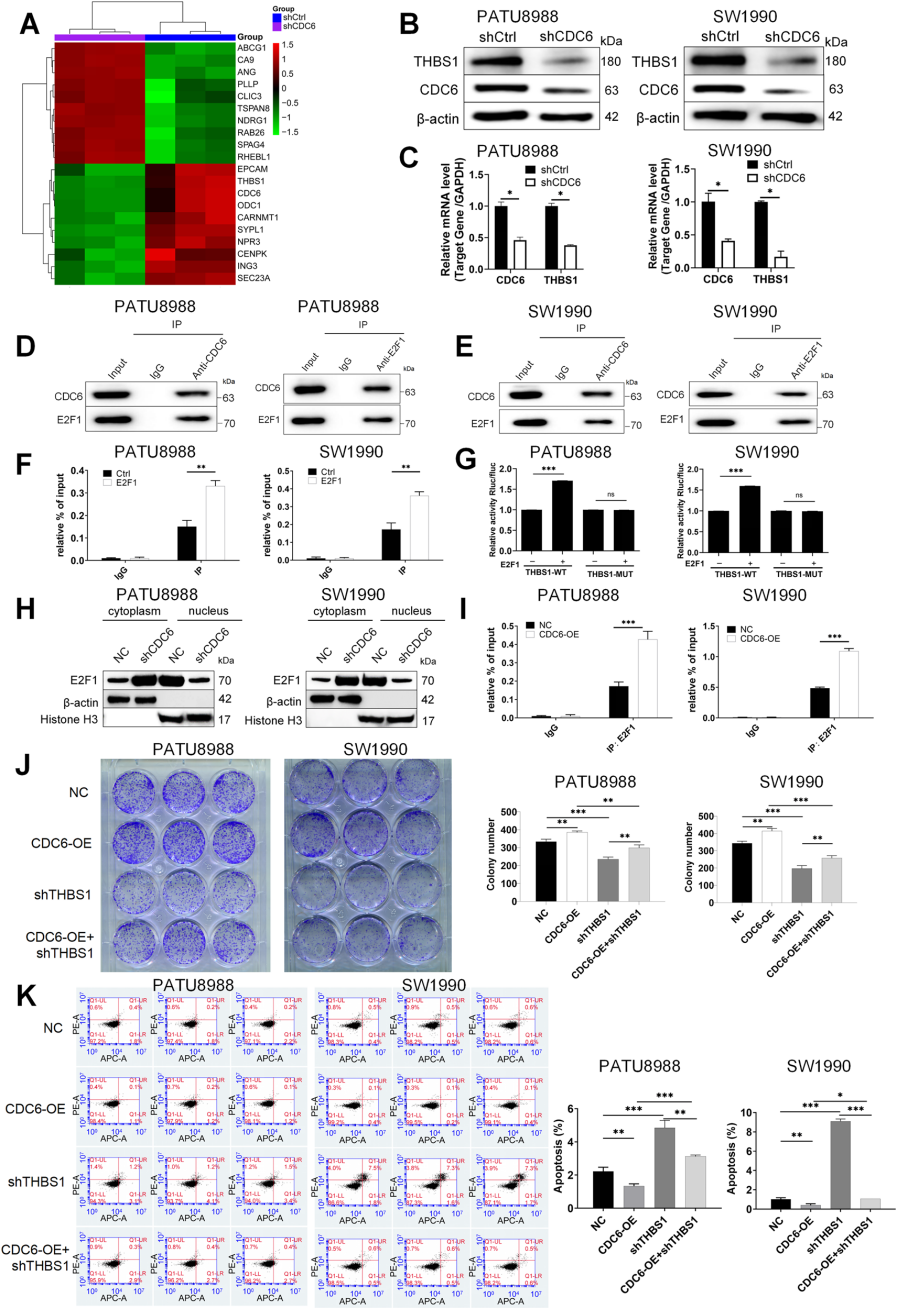
CDC6 promotes pancreatic cancer progression by upregulating THBS1 expression through interaction with E2F1. **A** Heat map depicting the expression of differential genes (The top 10 genes up-regulated or down-regulated) in three pairs of CDC6-knockdown pancreatic cancer cells and control group. Red and green indicate high and low gene expression. THBS1 suppression by CDC6 knockdown validated at (**B**) protein level through Western blot and **C** mRNA level through qPCR. **D**, **E** Reciprocal CDC6-E2F1 interaction demonstrated by co-immunoprecipitation in PATU8988 and SW1990 lysates. **F** ChIP-qPCR in E2F1-overexpressing cells showed more recruitment of E2F1 to the THBS1 promoter. **G** Dual-luciferase reporter assay confirmed E2F1 binding to THBS1-WT promoter, abolished in THBS1-MUT. **H** Subcellular fractionation revealed CDC6 knockdown reduces nuclear E2F1 and increases cytoplasmic retention through western blot. **I** CDC6 overexpression enhanced E2F1-THBS1 promoter binding in ChIP assays. **J** Colony formation assay showed the cell reproductive capacity enhanced by CDC6-OE, while reduced by THBS1 knockdown and exhibited intermediate ability in the rescue group. **K** Apoptosis of PATU8988 and SW1990 cells after overexpression of CDC6, knockdown of THBS1 and rescued processing was measured by flow cytometry. Data presented as mean ± SD. *P < 0.05, **P < 0.01, ***P < 0.001.

To investigate the molecular mechanism by which CDC6 promotes pancreatic cancer progression, genome-wide expression microarray analysis was conducted in CDC6-knockdown (shCDC6) versus control (shCtrl) PATU8988 cells, revealing THBS1 as a key downstream target of CDC6 (Fig. 3A). THBS1 expression was significantly downregulated at both protein (Figs. 3B and WB3A) and mRNA (Fig. 3C) levels following CDC6 knockdown. IHC staining of tissue microarrays (HPanA180Su03) further demonstrated elevated THBS1 expression in 50.6% of tumor tissues compared to 15.7% in adjacent normal tissues (*P* < 0.001, Table 4 and Supplementary Fig. S2A), with higher expression associated with advanced tumor stages and metastasis (*P* < 0.05; Tables 5, 6). Moreover, patients with high THBS1 expression exhibited reduced median survival (17.3 vs. 22.1 months, *P* < 0.05; Supplementary Fig. S2B), underscoring its prognostic significance. Bioinformatic analyses identified E2F1 as a transcription factor linking CDC6 to THBS1 regulation, supported by a high-confidence interaction between CDC6 and E2F1 (STRING score: 986/1000; Supplementary Table S1), and the presence of conserved E2F1 binding motifs in the THBS1 promoter.

**Table 4:** Expression patterns in pancreatic cancer tissues and para-carcinoma tissues were revealed in immunohistochemistry analysis.

THBS1 expression	Tumor tissue		Para-carcinoma tissue		*p* value
	Cases	Percentage	Cases	Percentage	
Low	40	49.4%	75	84.3%	<0.001
High	41	50.6%	14	15.7%

**Table 5:** Relationship between THBS1 expression and tumor characteristics in patients with pancreatic cancer.

Features	Cases	THBS1 expression		*p* value
		low	high	
All patients	81	40	41	
Age (years)				0.318
≤60	42	23	19	
>60	39	17	22	
Gender				0.442
Male	50	23	27	
Female	31	17	14	
Tumor size				0.754
<4 cm	56	27	29	
≥4 cm	25	13	12	
Stage				0.042
I	24	14	10	
II	26	16	10	
III	9	3	6	
IV	22	7	15	
T Infiltrate				0.408
T1	6	3	3	
T2	35	21	14	
T3	30	9	21	
T4	10	7	3	
Lymphaticmetastasis (N)				0.479
N0	29	17	12	
N1	42	17	25	
N2	10	6	4	
Metastasis				0.028
M0	60	34	26	
M1	21	6	15	
Degree of differentiation				0.171
low	4	0	4	
middle	68	35	33	
high	9	5	4	
Vasculature violations				0.567
low	46	24	22	
high	35	16	19	
Nerve bundle violations				0.876
low	27	13	14	
high	54	27	27

**Table 6:** Relationship between THBS1 expression and tumor characteristics in patients with pancreatic cancer.

	THBS1 expression (*n* = 81)	
	**Pearson correlation**	**Significance (two-tailed)**
Stage	0.227	0.041
Metastasis	0.246	0.027

Experimental validation confirmed that CDC6 interacts with E2F1 to regulate THBS1 expression. Co-immunoprecipitation (co-IP) assays demonstrated a direct interaction between CDC6 and E2F1 (Figs. 3D, E and WB3B, C). Chromatin immunoprecipitation (ChIP) assays showed increased E2F1 enrichment at the THBS1 promoter in E2F1-overexpressing PATU8988 and SW1990 cells (*P* < 0.01; Fig. 3F), while dual luciferase reporter assays indicated that E2F1 activates the wild-type THBS1 promoter but not its mutant form (*P* < 0.001; Fig. 3G). Western blot analyses revealed that CDC6 knockdown leads to a reduction of nuclear E2F1 levels, whereas CDC6 overexpression enhances E2F1’s binding to the THBS1 promoter (*P* < 0.001, Fig. 3H, I and WB4), suggesting that CDC6 may regulate the expression of THBS1 through transcriptional control mediated by E2F1.

To confirm the functional role of the CDC6/THBS1 regulatory axis, rescue experiments were performed using CDC6 overexpression (CDC6-OE) and THBS1 knockdown (shTHBS1). CDC6-OE significantly increased both CDC6 and THBS1 expression at mRNA and protein levels (*P* < 0.01), while shTHBS1 specifically reduced THBS1 expression without affecting CDC6 levels. Co-expression of CDC6-OE with shTHBS1 partially restored THBS1 expression (*P* < 0.01, vs CDC6-OE or shTHBS1), confirming the specificity of this regulatory axis (Supplementary Figs. S2C–F and WB5). Functionally, CDC6-OE enhanced colony formation (*P* < 0.01) and suppressed apoptosis (*P* < 0.01), whereas shTHBS1 inhibited clonogenic potential (*P* < 0.001) and promoted apoptosis (*P* < 0.001). Importantly, co-expression of CDC6-OE with shTHBS1 partially rescued these phenotypes, demonstrating that THBS1 mediates CDC6-driven tumorigenic effects (Fig. 3J, K).

Collectively, these results showed that CDC6 interacts with E2F1 to enhance its binding to the THBS1 promoter, thereby upregulating THBS1 expression and ultimately driving pancreatic cancer progression.

### CDC6 modulates pancreatic cancer’s malignant progression through glycolytic pathway regulation

Next, we performed integrated bioinformatics analyses on genome-wide transcriptional profiles from shCtrl and shCDC6 PATU8988 cells. KEGG pathway analysis revealed significant enrichment in glycolysis/gluconeogenesis and other metabolic pathways in CDC6-expressing cells (Supplementary Fig. S3A). Gene Set Enrichment Analysis further confirmed activation of the glycolysis pathway (Supplementary Fig. S3B).

**Fig. 4:**
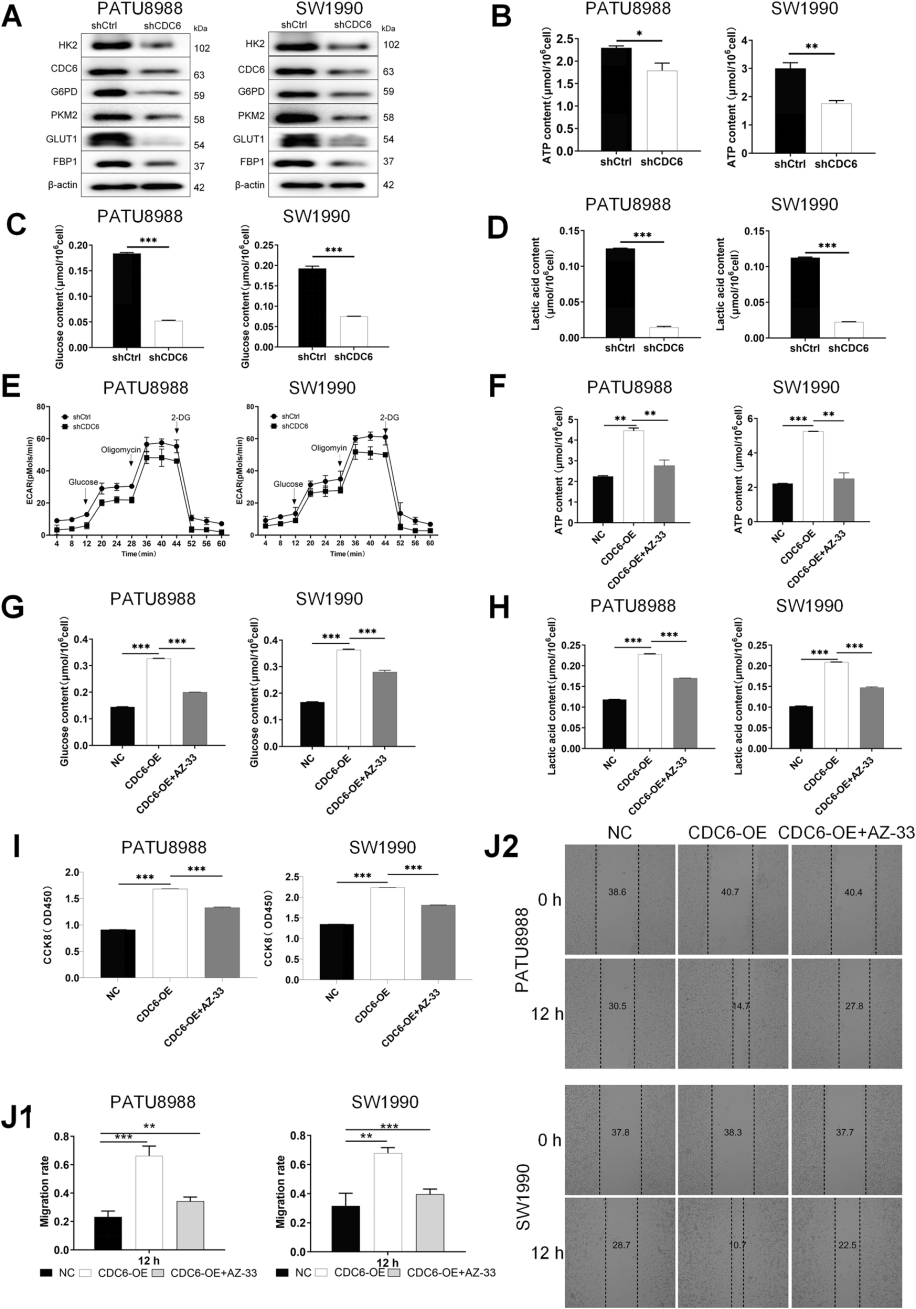
CDC6 drives pancreatic cancer oncogenic phenotypes through glycolysis. **A** Western blot analysis of glycolysis-associated proteins in CDC6-knockdown cells revealed significant downregulation of HK2, G6PD, PKM2, FBP1, and GLUT1. Metabolic profiling demonstrated CDC6 knockdown suppresses (**B**) ATP production, **C** Glucose consumption, **D** Lactate generation, **E** Extracellular acidification rate. ATP production (**F**), Glucose consumption (**G**), Lactate generation (**H**) were determined in PATU8988 and SW1990 cells treated with or without AZ-33 after CDC6 overexpression. **I** Cell viability was assessed by CCK-8 assay on PATU8988 and SW1990 cells treated with or without AZ-33 after CDC6 overexpression. **J**1–2. Migration potential evaluated by wound healing assay in PATU8988 and SW1990 cells treated with or without AZ-33 after CDC6 overexpression. Data expressed as mean ± SD. *P < 0.05, **P < 0.01, ***P < 0.001.

Western blot analysis showed that CDC6 knockdown downregulated key glycolytic enzymes hexokinase 2 (HK2) and glucose transporter 1 (GLUT1) (Figs. 4A and WB6). Metabolic assays showed reduced glycolytic flux in CDC6-deficient cells, with decreased intracellular ATP levels (*P* < 0.05 in PATU8988; *P* < 0.01 in SW1990; Fig. 4B), glucose consumption (*P* < 0.001; Fig. 4C), lactate production (*P* < 0.001; Fig. 4D), and extracellular acidification rate (ECAR) (Fig. 4E). We employed 2-deoxy-D-glucose (2-DG, 10 mM) to validate the correlation between changes in ECAR and glycolytic inhibition. CDC6-OE cells exhibited increased ECAR (*P* < 0.01), lactate production (*P* < 0.01) and decreased oxygen consumption rate‌ (OCR) (*P* < 0.01) compared to the negative control group (NC); 2-DG group exhibited decreased ECAR (*P* < 0.01), lactate production (*P* < 0.01) and increased OCR (*P* < 0.01) compared to the NC group; CDC6-OE cells pretreated with 2-DG exhibited no significant difference compared to the 2-DG control group (Supplementary Figs. S3–2, S3–3, S3–4).

To establish the causal relationship between CDC6-driven glycolysis and oncogenic phenotypes, we engineered CDC6-OE pancreatic cancer cells and inhibited glycolysis with AZ-33 (10 μM, Selleck), a selective HK2 inhibitor. CDC6-OE cells exhibited increased ATP production (*P* < 0.01; Fig. 4F), glucose uptake (*P* < 0.001; Fig. 4G), and lactate production (*P* < 0.001; Fig. 4H) compared to the negative control group (NC). While the glycolytic effects of CDC6-OE were partially abrogated by pharmacological inhibition of HK2 using AZ-33, indicating a role for CDC6 in metabolic regulation. Functional validation through CCK-8 assays revealed that CDC6-OE enhanced cell growth at 24 h (*P* < 0.001), which was reversed by AZ-33 (*P* < 0.001) (Fig. 4I). Wound healing assays demonstrated CDC6-OE accelerated wound closure at 12 h (PATU8988, *P* < 0.001; SW1990, *P* < 0.01), an effect significantly attenuated by AZ-33 (PATU8988, *P* < 0.01; SW1990, *P* < 0.001) (Fig. 4J1–J2). These findings conclusively demonstrate that CDC6 drives pancreatic cancer progression by enhancing glycolytic metabolism.

### CDC6 orchestrates pancreatic cancer malignant progression and glycolysis via THBS1-mediated signaling and AKT pathway activation

Literature review indicates that the activation of the PI3K/AKT pathway in pancreatic cancer enhances the migration and proliferation of pancreatic cancer cells [28, 29] and promotes aerobic glycolysis through the regulation of glycolysis-related genes [30]. At the same time, we found that in studies of prostate cancer and nasopharyngeal cancer, specific signaling axes may exist, enabling CDC6 or THBS1 to regulate the AKT signaling pathway, thereby influencing tumor proliferation and glycolysis [31, 32]. Based on these findings, we propose that CDC6 mechanistically promotes pancreatic cancer progression by orchestrating THBS1-mediated activation of the AKT pathway, thereby driving aerobic glycolytic reprogramming and sustaining oncogenic survival.

**Fig. 5:**
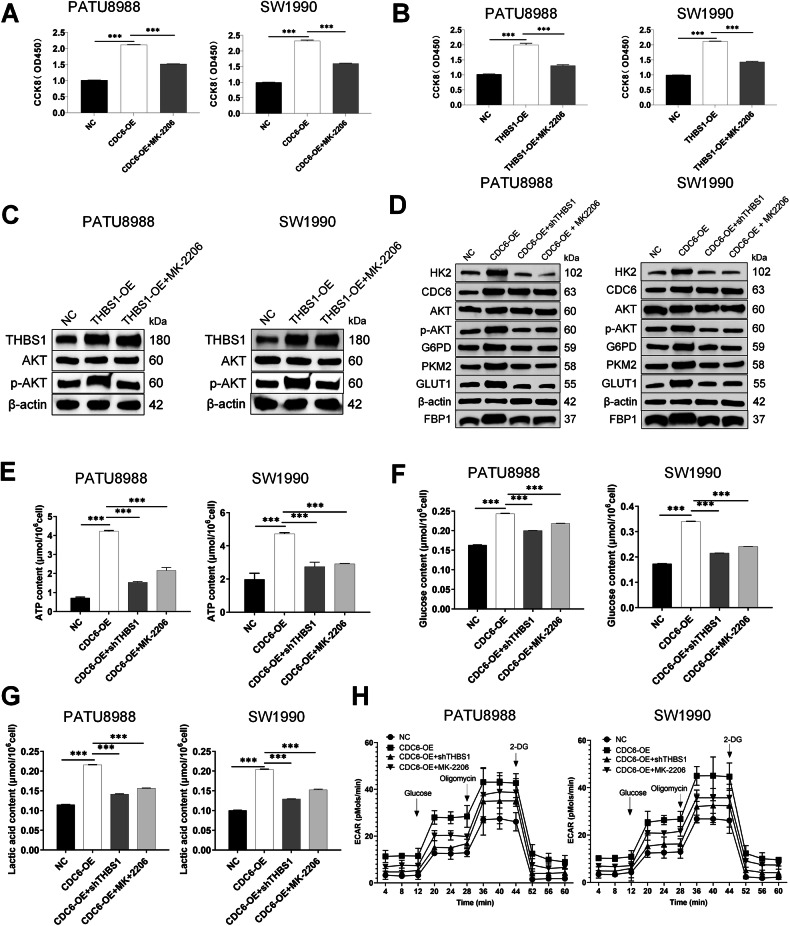
CDC6 orchestrates pancreatic cancer malignant progression and glycolysis via THBS1-mediated signaling and AKT pathway activation. **A** Cell viability assessed by CCK-8 assay on PATU8988 and SW1990 cells treated with or without MK-2206 after CDC6 overexpression. **B** Cell viability assessed by CCK-8 assay on PATU8988 and SW1990 cells treated with or without MK-2206 after THBS1 overexpression. **C** Western blot analysis of p-AKT and AKT protein levels in PATU8988 and SW1990 cells with or without MK-2206 after THBS1 overexpression. **D** Western blot analysis of p-AKT and glycolytic enzymes in PATU8988 and SW1990 cells with or without THBS1 knockdown or MK-2206 after CDC6 overexpression. Metabolic assays showed CDC6-OE increased (**E**) ATP production, **F** Glucose consumption, **G** Lactate generation, **H** ECAR in PATU8988 and SW1990 cells, while THBS1 depletion or AKT inhibition reversed these effects. Data represent mean ± SD. **P < 0.01, ***P < 0.001.

To mechanistically investigate the relationship between THBS1 and AKT, we performed genetic and pharmacological perturbations in two pancreatic cancer models. Western blot analysis of THBS1-overexpressing (THBS1-OE) cells demonstrated an increase in THBS1 protein expression level (*P* < 0.001) and in AKT phosphorylation (p-AKT/AKT ratio; *P* < 0.001) compared to the NC group, while total AKT levels remained comparable (Figs. 5C and WB7). Strikingly, treatment with the AKT inhibitor MK-2206 (10 μM, Selleck) in THBS1-OE cells reduced p-AKT levels (*P* < 0.01 vs THBS1-OE alone) without altering total AKT expression.

To investigate the hierarchical signaling of CDC6/THBS1/AKT in functional role, we engineered CDC6-OE pancreatic cancer cells (CDC6-OE group), CDC6-OE with THBS1 knockdown cells (CDC6-OE+shTHBS1 group), Negative control pancreatic cancer cells (NC group), and inhibited AKT pathway with MK-2206 (10 μM, Selleck), an allosteric AKT inhibitor (CDC6-OE + MK-2206 group). Western blot analysis revealed that CDC6-OE upregulated key glycolytic enzymes, such as HK2 and GLUT1 (*P* < 0.01), concomitant with enhanced AKT phosphorylation compared to NC (*P* < 0.01) (Figs. 5D and WB8). Notably, THBS1 depletion or AKT inhibition abrogated these effects (vs CDC6-OE; *P* < 0.01). CCK-8 assays demonstrated that CDC6-OE enhanced proliferation at 24 h compared to vector controls (P < 0.001), whereas MK-2206 co-treatment reduced this proliferative effect (*P* < 0.001 vs CDC6-OE) (Fig. 5A). Wound healing assays revealed CDC6-OE accelerated migration at 12 h (*P* < 0.001), an effect significantly attenuated by MK-2206 (*P* < 0.01) (Supplementary Fig. S4A, B).

To delineate the THBS1/AKT signaling axis in pancreatic cancer progression, we generated THBS1-overexpressing (THBS1-OE) cells and pharmacologically inhibited AKT using MK-2206. CCK-8 assays demonstrated THBS1-OE enhanced cell viability at 24 h compared to control group (*P* < 0.001), whereas MK-2206 co-treatment reduced this effect by (*P* < 0.001) (Fig. 5B). Flow cytometry revealed THBS1-OE suppressed apoptosis relative to control group (*P* < 0.001), the anti-apoptotic phenotype reversed by MK-2206 (*P* < 0.001) (Supplementary Fig. S4C, D).

Functional metabolic assays demonstrated that CDC6-OE augmented ATP production (*P* < 0.001), Glucose consumption(*P* < 0.001), Lactate production(*P* < 0.001), ECAR (*P* < 0.001), reduced OCR (*P* < 0.01), compared to NC group. These glycolytic phenotypes were substantially attenuated in CDC6-OE+shTHBS1 groups and CDC6-OE + MK-2206 groups (*P* < 0.001) (Fig. 5E–H and Supplementary Figs. S4–2). Collectively, these findings demonstrate that CDC6 orchestrates oncogenic glycolysis and malignant progression in pancreatic cancer through a hierarchical THBS1/AKT signaling cascade.

### CDC6 modulates glycolysis and malignant progression in vivo via thbs1 in pancreatic cancer

To validate the CDC6/THBS1/AKT axis in vivo, we established subcutaneous xenograft models using pancreatic cancer cells with four genetic configurations: Negative control (NC); THBS1 knockdown (shTHBS1); CDC6 overexpression (CDC6-OE group); CDC6-OE with shTHBS1 (CDC6-OE+ shTHBS1 group).

**Fig. 6:**
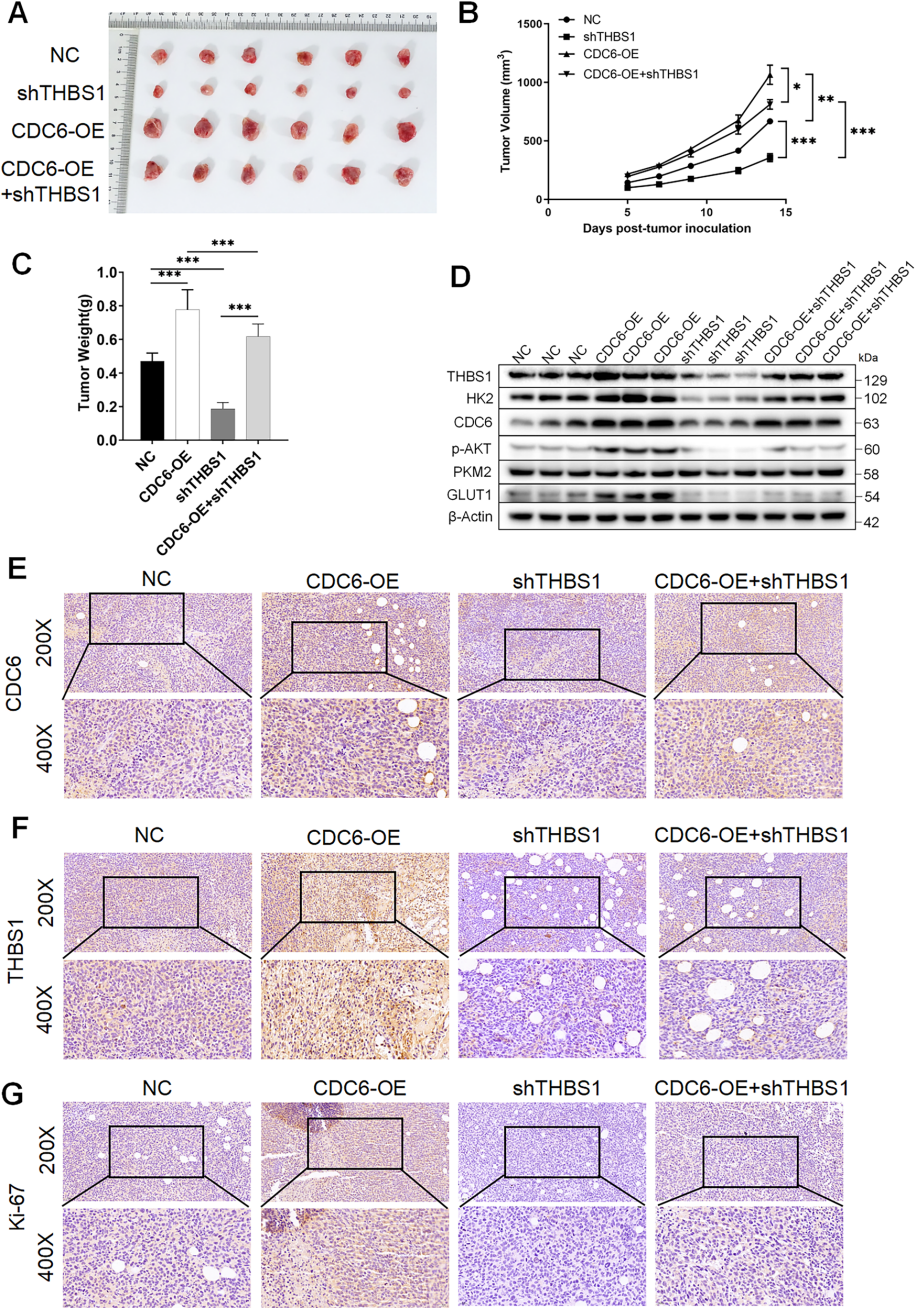
CDC6 modulates glycolysis and malignant progression in vivo via THBS1 in pancreatic cancer. **A** 1 × 10^7^ NC or shTHBS1 or CDC6-OE or shTHBS1 + CDC6-OE PATU8988 cells were injected into the armpit of 6-week-old female NCG mice to induce a xenograft tumor. Tumor images from four experimental groups (*n* = 6) at the end of the experiment are shown. **B** Tumor growth curves were made by measuring the tumor volume between 14 days after injection. **C** Mice were sacrificed at day 15 post-injection, and the final tumor weight was measured in each group. **D** Western blot analysis of glycolytic enzymes and p-AKT levels in each group. IHC staining was performed in mouse tumor tissues to clarify the expressions of (**E**) CDC6, **F** THBS1, and **G** KI67. Data expressed as mean ± SD. *P < 0.05, **P < 0.01, ***P < 0.001.

In vivo functional validation of the CDC6/THBS1 axis revealed profound effects on tumor growth dynamics (Fig. 6A–C). Subcutaneous xenografts in the shTHBS1 group exhibited a reduction in final tumor volume (*P* < 0.001) and a decrease in tumor weight (*P* < 0.001) compared to NC, whereas CDC6-OE tumors demonstrated volume expansion (*P* < 0.01) and weight gain (*P* < 0.001) vs NC. The rescue cohort (CDC6-OE+shTHBS1) displayed partial phenotype reversal, in tumor volume (vs CDC6-OE, *P* < 0.05; vs shTHBS1, *P* < 0.001) and tumor mass (*P* < 0.001), confirming THBS1 dependency. Western blot of tumor lysates demonstrated CDC6-OE upregulated glycolytic markers (GLUT1, HK2, vs NC, *P* < 0.01), signaling components (THBS1, p-AKT, vs NC, *P* < 0.01). These effects were abolished in shTHBS1 tumors (*P* < 0.001) and partially rescued in CDC6-OE+shTHBS1 (*P* < 0.05) (Figs. 6D and WB9). IHC revealed distinct expression patterns across experimental cohorts (Fig. 6E–G): The expressions of CDC6 (*P* < 0.01), THBS1 (*P* < 0.01), and Ki-67 (*P* < 0.001) were significantly upregulated in the transplanted tumor tissues of the CDC6-OE group compared to the NC group. In the transplanted tumor of the shTHBS1 group, the expressions of THBS1 (*P* < 0.001), and Ki-67 (*P* < 0.01) were significantly decreased compared to NC group, while CDC6 levels showed no significant change. CDC6+shTHBS1 group demonstrated that CDC6 levels were comparable to CDC6-OE alone (*P* > 0.05), partial restoration of THBS1 (*P* < 0.01) and intermediate levels of Ki-67 (*P* < 0.01).

To supplement the validation of the CDC6/THBS1/AKT axis in vivo, we established subcutaneous xenograft models using pancreatic cancer cells with four treatment methods: Negative control (NC); CDC6 overexpression (CDC6-OE group); CDC6-OE with MK-2206 injection (CDC6-OE+MK-2206 group); CDC6-OE with 2-DG injection (CDC6-OE+2-DG group).

In vivo functional validation of the axis revealed profound effects on tumor growth dynamics (Supplementary Fig. S5A–C). Subcutaneous xenografts in the CDC6-OE tumors demonstrated volume expansion (*P* < 0.001) and weight gain (*P* < 0.001) compared to NC. The CDC6-OE + MK-2206 and CDC6-OE + 2-DG group displayed partial phenotype reversal, in tumor volume (vs CDC6-OE, *P* < 0.01) and tumor weight (*P* < 0.01), confirming AKT and glycolytic dependency. Western blot of tumor lysates demonstrated CDC6-OE upregulated glycolytic markers (GLUT1, HK2, vs NC, *P* < 0.01), signaling components (THBS1, p-AKT, vs NC, *P* < 0.01). Glycolytic markers and p-AKT were rescued in CDC6-OE + MK-2206 and CDC6-OE + 2-DG group (*P* < 0.05) (Supplementary Figs. S5D and WB10). IHC revealed distinct expression patterns across experimental cohorts (Fig. S5E): The expressions of Ki-67 (*P* < 0.01) were significantly upregulated in the transplanted tumor tissues of the CDC6-OE group compared to the NC group. In the transplanted tumor of the CDC6-OE + MK-2206 and CDC6-OE + 2-DG group, the expressions of Ki-67 (*P* > 0.05) were not significantly decreased compared to NC group.

Collectively, these findings confirm in vivo that CDC6 promotes glycolysis and malignant progression of pancreatic cancer via THBS1-mediated mechanisms.

## Discussion

CDC6 plays a pivotal role in orchestrating eukaryotic DNA replication initiation. Its dysregulated overexpression is recognized as both a diagnostic hallmark and pathogenic driver in multiple malignancies. A study employed an integrated approach that combined pan-cancer analysis with machine learning modeling to investigate the potential of CDC6 as a prognostic biomarker and a therapeutic target for immunotherapy [33]. Experimental evidence demonstrates CDC6’s oncogenic potential across tumor types: In osteosarcoma models, targeted CDC6 suppression significantly reduces cellular proliferation and invasiveness while inducing apoptosis [34]; Similarly, breast cancer specimens exhibit elevated CDC6 levels that demonstrate positive correlation with clinical staging and promote tumor cell proliferation/metastasis [35]; This overexpression pattern extends to retinoblastoma, non-small cell lung carcinoma, and gliomas [14]. However, a paradoxical tumor suppressor effect has been reported in prostate cancer models, where CDC6 overexpression unexpectedly inhibits cell proliferation, potentially due to tissue-specific characteristics [15]. In this study, we identified that CDC6 is significantly upregulated in pancreatic cancer tissues. Moreover, the overexpression of CDC6 is closely associated with tumor staging and metastatic progression. Patients with high CDC6 expression exhibited significantly shortened overall survival and progression-free survival. In vitro experiments demonstrated that the proliferation, clonal formation, migration, and invasion capabilities of pancreatic cancer cells were markedly impaired upon CDC6 inhibition, while apoptosis was increased. These findings collectively suggest that CDC6 plays a critical role in promoting the malignant behavior of pancreatic cancer cells and its potential as a prognostic marker or therapeutic target.

THBS1 serves as a master regulator governing multiple fundamental biological processes, ranging from intercellular communication and extracellular matrix dynamics to angiogenesis and inflammatory regulation [36–38]. Emerging evidence highlights its context-dependent oncogenic functions across malignancies. For example, in gastric cancer, researchers have uncovered an FN1 3’-UTR/let-7i-5p/THBS1 regulatory axis that potentiates tumor cell invasion and metastatic dissemination [39]. Breast cancer studies reveal a mechanotransduction pathway where YAP-mediated THBS1 overexpression activates FAK signaling to enhance tumor cell adhesion and local invasion [40]. Similar pro-tumorigenic roles have been documented in colorectal adenocarcinoma and oral squamous cell carcinoma through distinct molecular mechanisms [41, 42]. In pancreatic cancer, previous studies have demonstrated that knockdown of THBS1 can suppress tumor progression and induce apoptosis, potentially through inhibition of the JAK2/STAT3 signaling pathway [43]. Notably, one study has shown that hsa_circ_0007919 significantly promotes tumor cell migration, invasion, and lung metastasis by recruiting Sp1 to suppress THBS1 transcription [44]. These findings appear to contrast with certain aspects of our results. The discrepancies may arise from methodological and material differences between the studies. At the clinical tissue level, our analysis relied on tissue microarrays, whereas the comparator study utilized bulk clinical specimens. Such distinctions may introduce unintended biases; the subsequent use of divergent cell lines could further amplify these discrepancies following experimental intervention. These findings suggest that THBS1 may be involved in a more intricate regulatory network in pancreatic cancer pathogenesis. Future studies incorporating a broader range of cell lines and larger prospective cohorts would enhance robustness and generalizability. In our study, whole-genome expression microarray analysis identified THBS1 as a gene that was significantly down-regulated following CDC6 knockdown. Additionally, its elevated expression in tumor tissues was found to be associated with an unfavorable prognosis.

As the fundamental process of RNA synthesis from DNA templates, transcription enables genetic information transfer to functional RNA species. Within this framework, E2F1—a cell cycle-associated transcription factor—demonstrates conserved interaction with CDC6 across physiological and neoplastic contexts [15, 45]. Mechanistic studies further reveal CDC6’s signaling integration through KRAS mutation-associated pathways, where it potentially mediates epigenetic silencing of CDH1 tumor suppressor via transcriptional repression [46]. Furthermore, CDC6 facilitates cell cycle progression through cyclin E-CDK2 complex binding, inducing CCNE1 transcriptional hyperactivity that drives uncontrolled proliferation [47]. Building on this mechanistic framework, our study elucidates CDC6’s novel transcriptional control over THBS1 via E2F1-mediated regulation, thereby expanding the known repertoire of CDC6-dependent oncogenic pathways. This discovery of the CDC6/E2F1/THBS1 transcriptional axis not only corroborates prior observations of CDC6’s gene regulatory capacity but also uncovers a therapeutically exploitable node at the intersection of cell cycle control and metabolic reprogramming in pancreatic cancer.

We conducted a comprehensive series of functional validation experiments to confirm the promoting role of the CDC6/THBS1 axis in pancreatic cancer progression. In addition, we reviewed the literature and identified that the glycolysis level in pancreatic cancer cells is significantly higher than that in normal cells, exhibiting a positive correlation with malignant phenotypes, including proliferation and migration capabilities [23]. Our experimental data revealed that genetic knockdown of CDC6 significantly downregulated pivotal glycolytic enzymes and reduced glycolytic flux. Conversely, overexpression of CDC6 enhanced glycolytic capacity, which was quantitatively attenuated upon treatment with pharmacological glycolysis inhibitors. These results suggest that CDC6 plays a critical role in driving pancreatic cancer progression through the regulation of glycolytic flux. In pancreatic ductal adenocarcinoma, studies have demonstrated that pancreatic cancer cells may activate the PI3K/Akt signaling pathway via the cFAM124A/Cathepsin L/tRXRα or FTO/USP7/NEDD4/PTEN axis, thereby contributing to tumor-mediated drug resistance [48, 49]. Also, PI3K/Akt/mTOR pathway activation induces c-Myc and HIF-1α stabilization, resulting in hyperactive glycolytic flux that sustains tumorigenesis [26]. Parallel investigations in hepatocellular carcinoma demonstrate this pathway’s conserved role in potentiating aerobic glycolysis to accelerate tumor growth and metastatic dissemination [25]. The findings of our study further confirmed that both CDC6 and THBS1 exert regulatory effects on the AKT signaling pathway. Our study also demonstrated that CDC6 co-regulates aerobic glycolysis and tumor progression via dysregulation of the AKT pathway mediated by THBS1. The influence of the CDC6/THBS1/AKT axis on tumor growth and glycolysis-related proteins was confirmed through in vivo experiments.

There are certain limitations to this experiment that warrant consideration. The xenograft tumor model differs from the actual in vivo environment of the human body, and therefore, the results may not fully represent tumor growth dynamics in humans. Additionally, the sample size of the animal experiments could be expanded for greater statistical power. In the cell assays, only two cell lines were utilized, which cannot comprehensively reflect the behavior of all pancreatic cancer cell lines; further validation using additional cell lines is necessary. Lastly, the public database employed in this study will be updated in the future, and the findings should be re-evaluated with the latest dataset to ensure their robustness.

In this study, we systematically investigated the oncogenic role of CDC6 in pancreatic cancer and its underlying molecular mechanisms. Our findings demonstrated that CDC6 was significantly upregulated in pancreatic cancer and correlated with advanced tumor stages, metastatic progression, and reduced patient survival. Mechanistically, we identified that CDC6 enhanced THBS1 promoter activity through interaction with E2F1. Importantly, our data further revealed that CDC6 collaborated with THBS1-mediated AKT signaling to modulate the glycolytic pathway, thereby facilitating pancreatic cancer progression.

## Conclusion

Collectively, these results indicate that CDC6 synergistically promotes pancreatic cancer progression via the THBS1-AKT signaling axis and metabolic reprogramming, highlighting the CDC6/THBS1/AKT axis as a potential therapeutic target for this highly aggressive malignancy.

## Supplementary information

Supplementary results

Table S1

Supplementary Tables

Supplementary material for WB

## Data Availability

The data generated in this study are available upon request from the corresponding author.

## Code availability

The computer code used in this study is available upon request from the corresponding author.
