# Supplementary material for: CDC6/THBS1 accelerates pancreatic cancer progression via AKT-mediated glycolytic reprogramming
Source: Cell Death Dis. 2026 Apr 21;17(1):524. doi: 10.1038/s41419-026-08758-2 (PMC13230735; doi:10.1038/s41419-026-08758-2)
Supplement: Supplementary file 1 — Supplementary results [file 41419_2026_8758_MOESM1_ESM.docx]

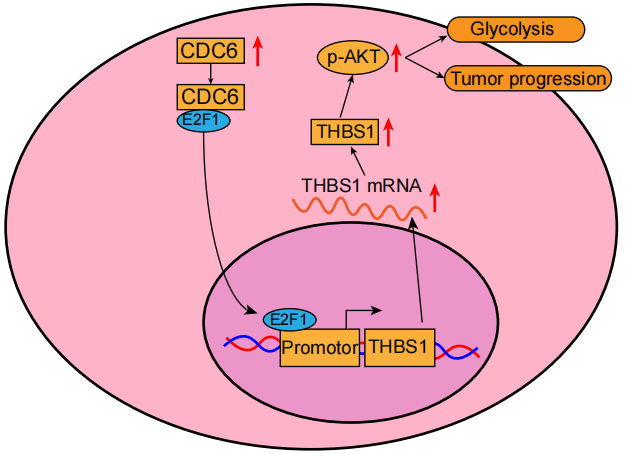


**Figure S.** Schematic diagram of the molecular mechanism of this study.


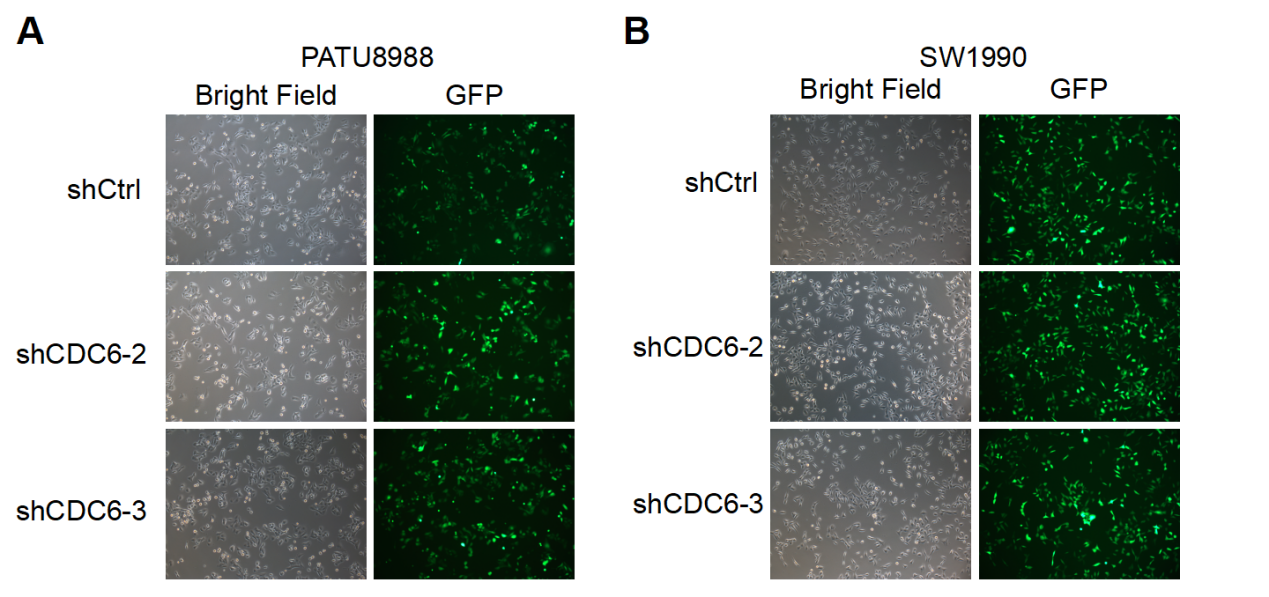


**Figure S1. Construct CDC6 knockdown cell lines. A.** CDC6-knockdown pancreatic cancer cell line PATU8988 was constructed by lentivirus transfection. **B.** CDC6-knockdown pancreatic cancer cell line SW1990 was constructed by lentivirus transfection.


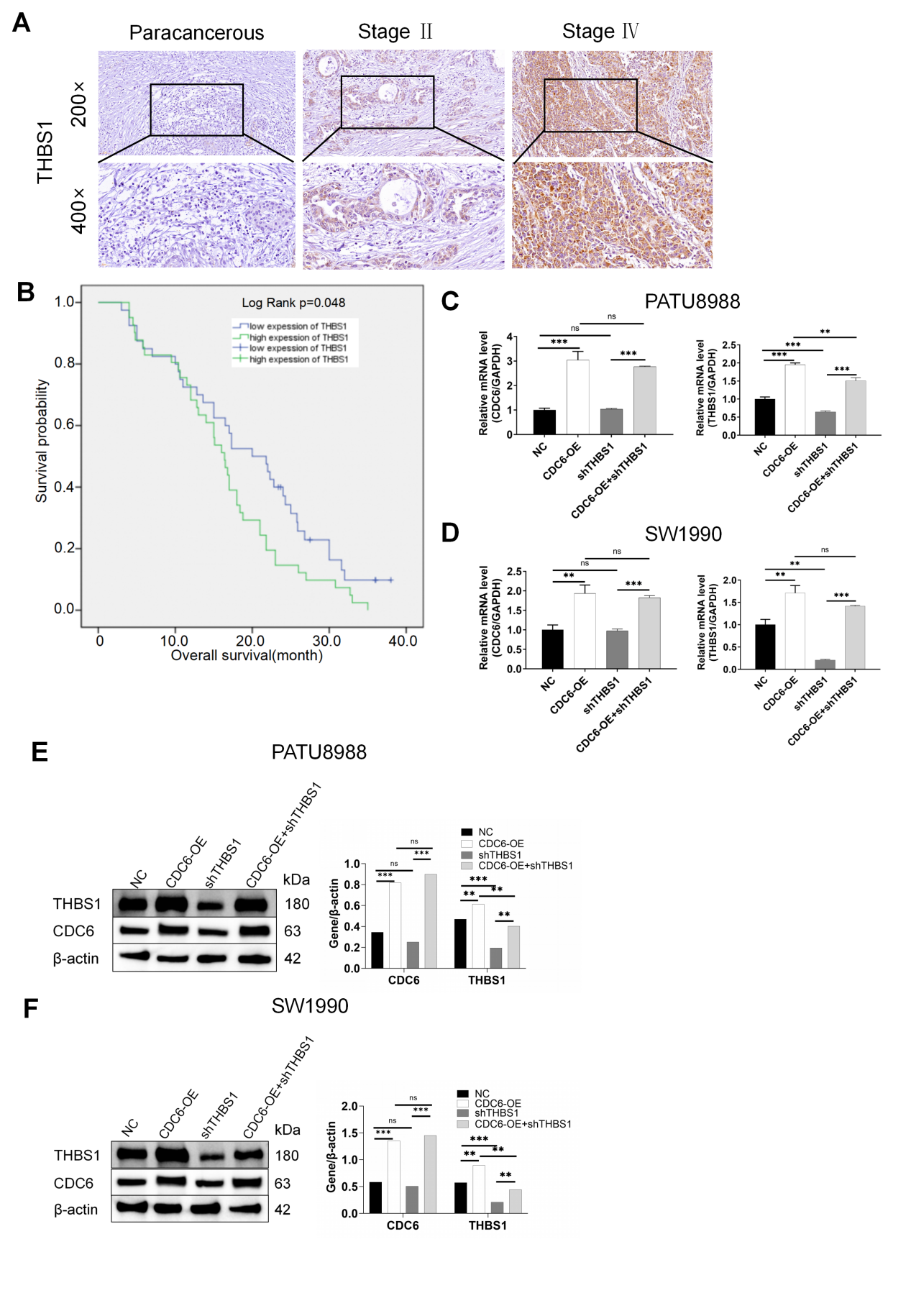


**Figure S2. Identification of downstream molecules of CDC6 and study of regulatory mechanisms. A.** IHC results of THBS1 in pancreatic cancer tissue microarray containing adjacent tissues. **B.** KM curve of overall survive of patients with different expressions of THBS1 in the tissue microarray. **C, D.** With CDC6 overexpression or THBS1 knockdown, the expression of CDC6 and THBS1 mRNA in PATU8988 and SW1990 was verified by qPCR. **E, F.** With CDC6 overexpression or THBS1 knockdown, verifying the expression of CDC6 and THBS1 proteins in PATU8988 and SW1990 through WB. **Data expressed as mean ± SD. *P<0.05, **P<0.01, ***P<0.001.**


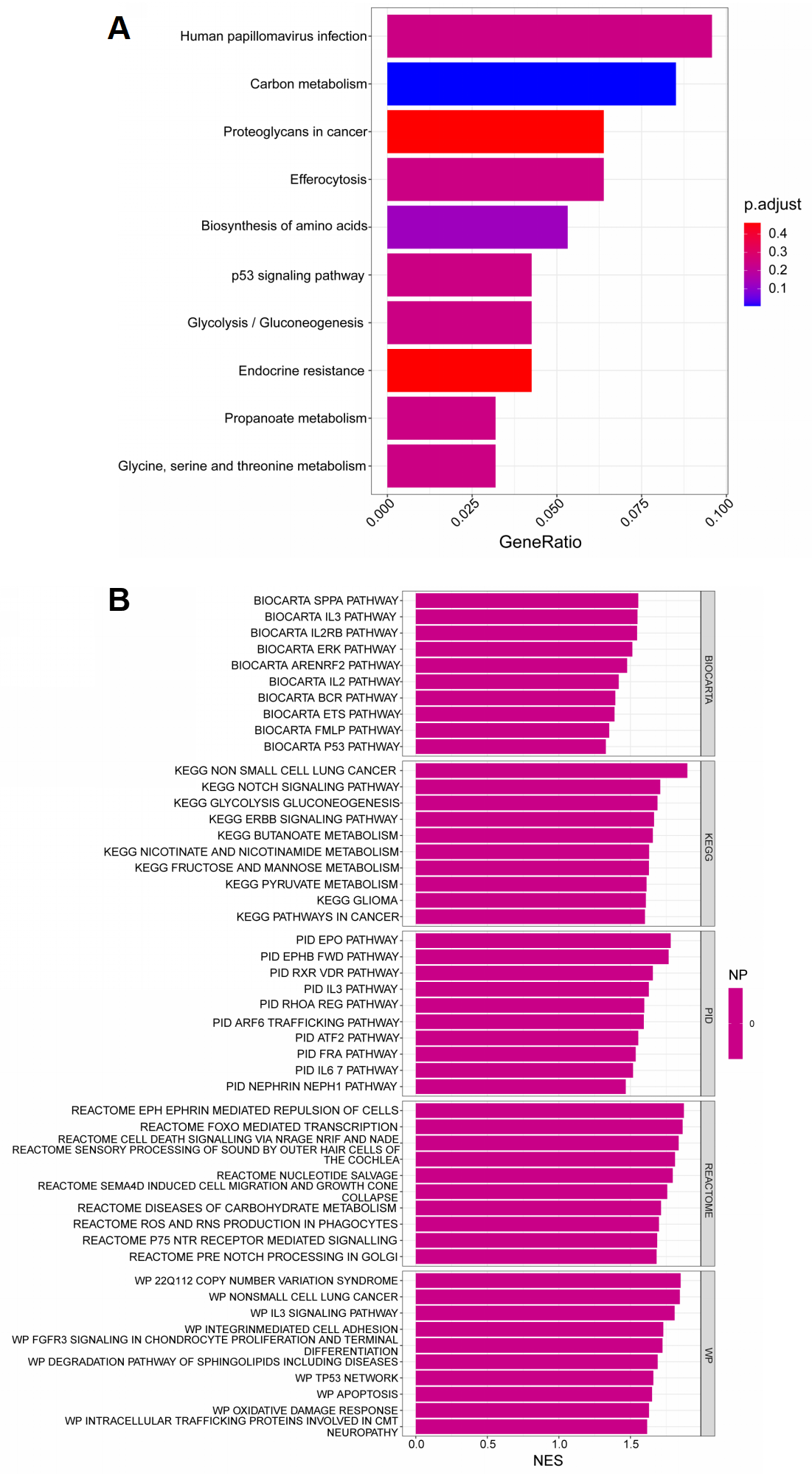


**Figure S3. CDC6 affects tumor cell phenotype through glycolysis. A.** KEGG enrichment analysis (top 10 pathways) of genes co‑expressed with CDC6. **B.** The GSEA software was utilized to conduct classical pathway enrichment analysis. Screening for significantly enriched genes in the top ten sets of classical pathways from KEGG, BIOCARD, PID, REACTOME, and WIKIPATHWAYS.


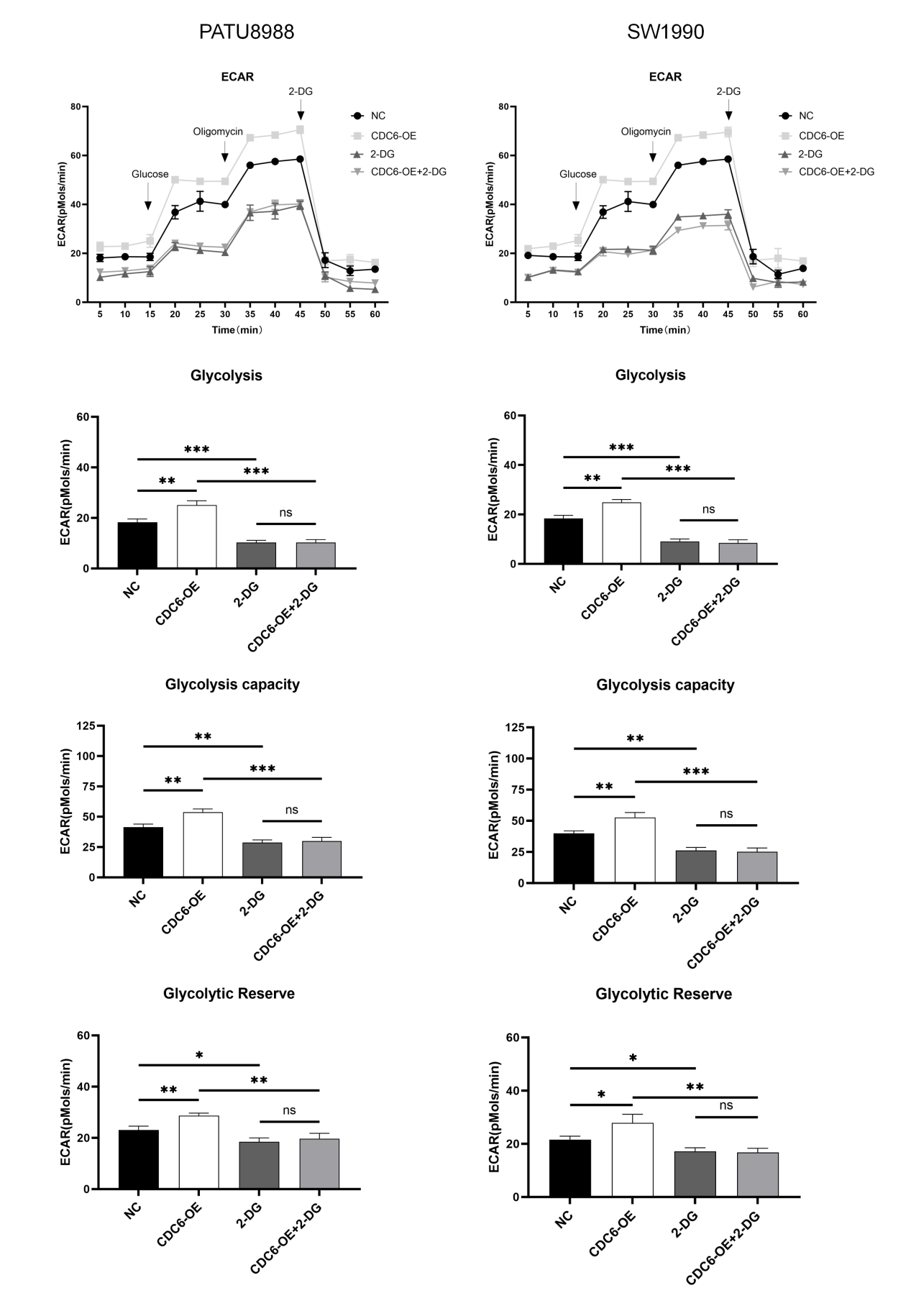


**Figure S3-2. CDC6 affects tumor cell phenotype through glycolysis.**  Metabolic assays showed CDC6-OE increased ECAR in PATU8988 and SW1990 cells, while 2-DG decreased ECAR and reversed these effects. **Data expressed as mean ± SD. *P<0.05, **P<0.01, ***P<0.001.**

**
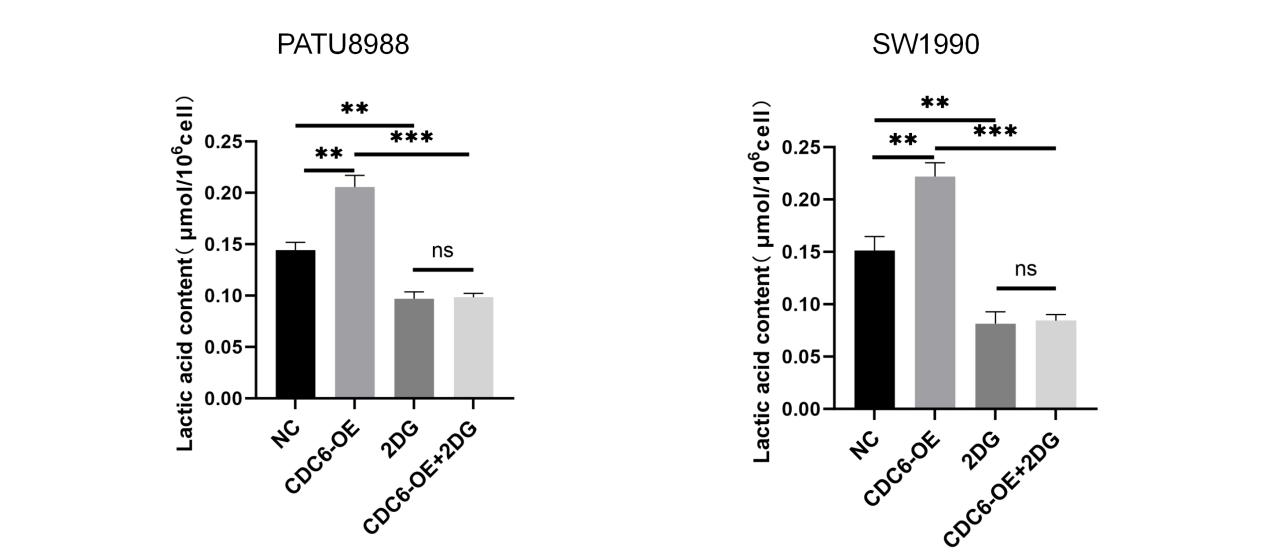
**

**Figure S3-3. CDC6 affects tumor cell phenotype through glycolysis.** Metabolic assays showed CDC6-OE increased Lactate generation in PATU8988 and SW1990 cells, while 2-DG decreased lactate generation and reversed these effects. **Data expressed as mean ± SD. *P<0.05, **P<0.01, ***P<0.001.**

**
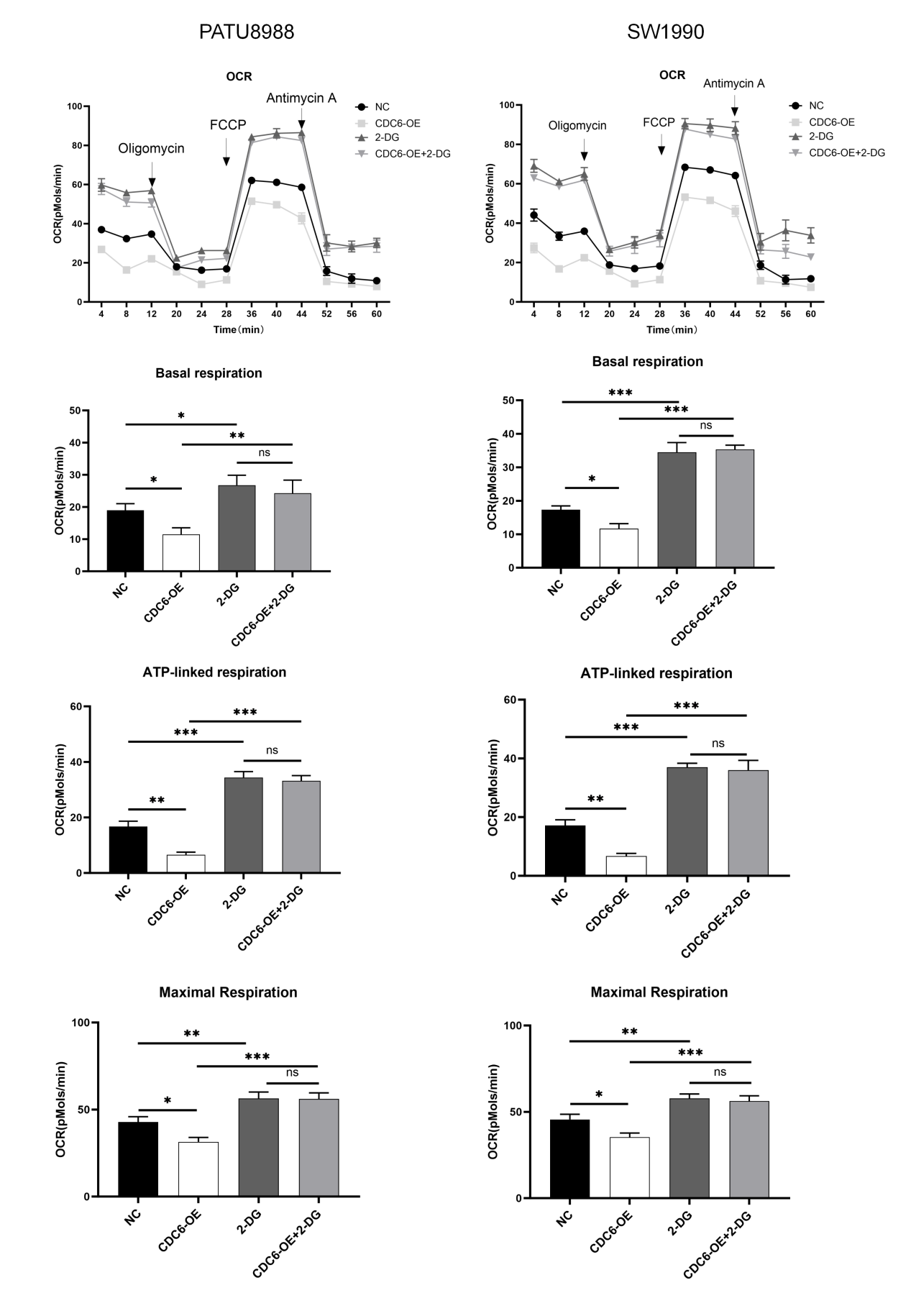
**

**Figure S3-4. CDC6 affects tumor cell phenotype through glycolysis.** Metabolic assays showed CDC6-OE decreased OCR in PATU8988 and SW1990 cells, while 2-DG increased OCR and reversed these effects. **Data expressed as mean ± SD. *P<0.05, **P<0.01, ***P<0.001.**

**
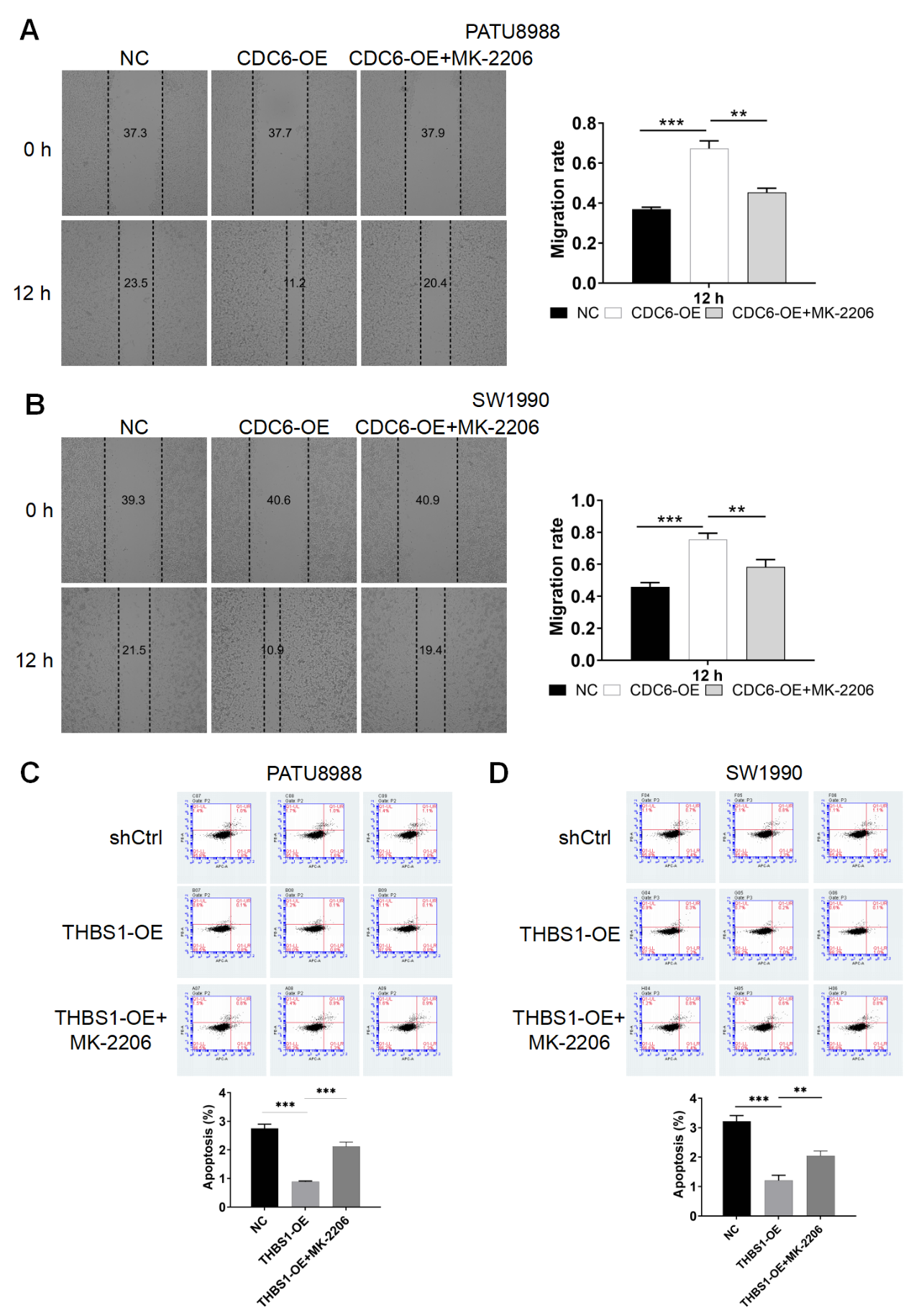
**

**Figure S4. CDC6 regulates pancreatic cancer progression through the AKT signaling pathway. A, B.** Wound-healing assays were performed to investigate migration ability after overexpressing CDC6 in PATU8988 and SW1990 cells treated with or without MK-2206. **C, D.** Differences in cell apoptosis were detected by flow cytometry after overexpressing THBS1 in PATU8988 and SW1990 cells treated with or without MK-2206. **Data expressed as mean ± SD. *P<0.05, **P<0.01, ***P<0.001.**

**
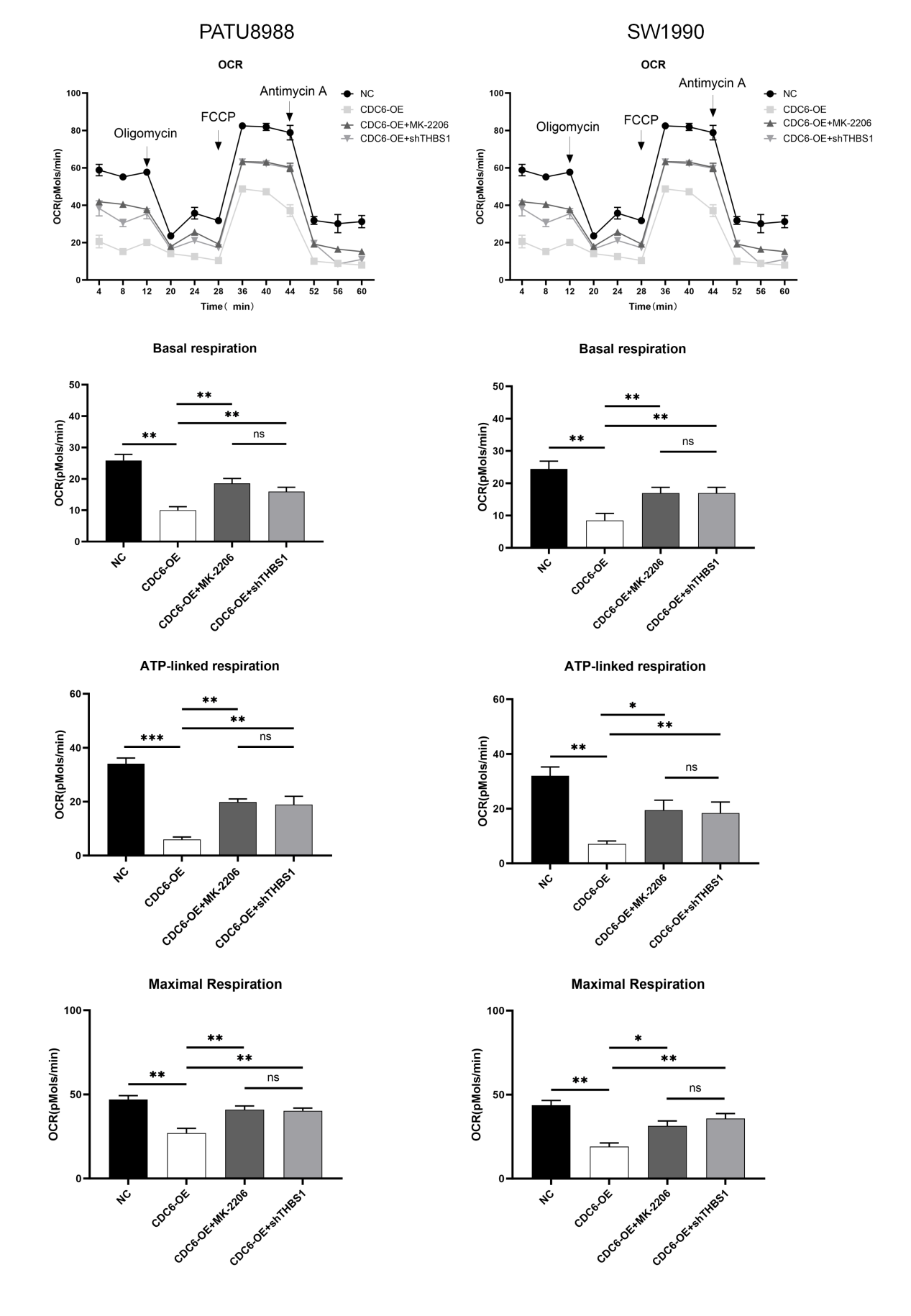
**

**Figure S4-2. CDC6 regulates pancreatic cancer progression through THBS1-mediated signaling and AKT pathway activation.** Metabolic assays showed CDC6-OE decreased OCR in PATU8988 and SW1990 cells, while THBS1 depletion or AKT inhibition reversed these effects. **Data represent mean ± SD. *P<0.05, **P<0.01, ***P<0.001.**

**
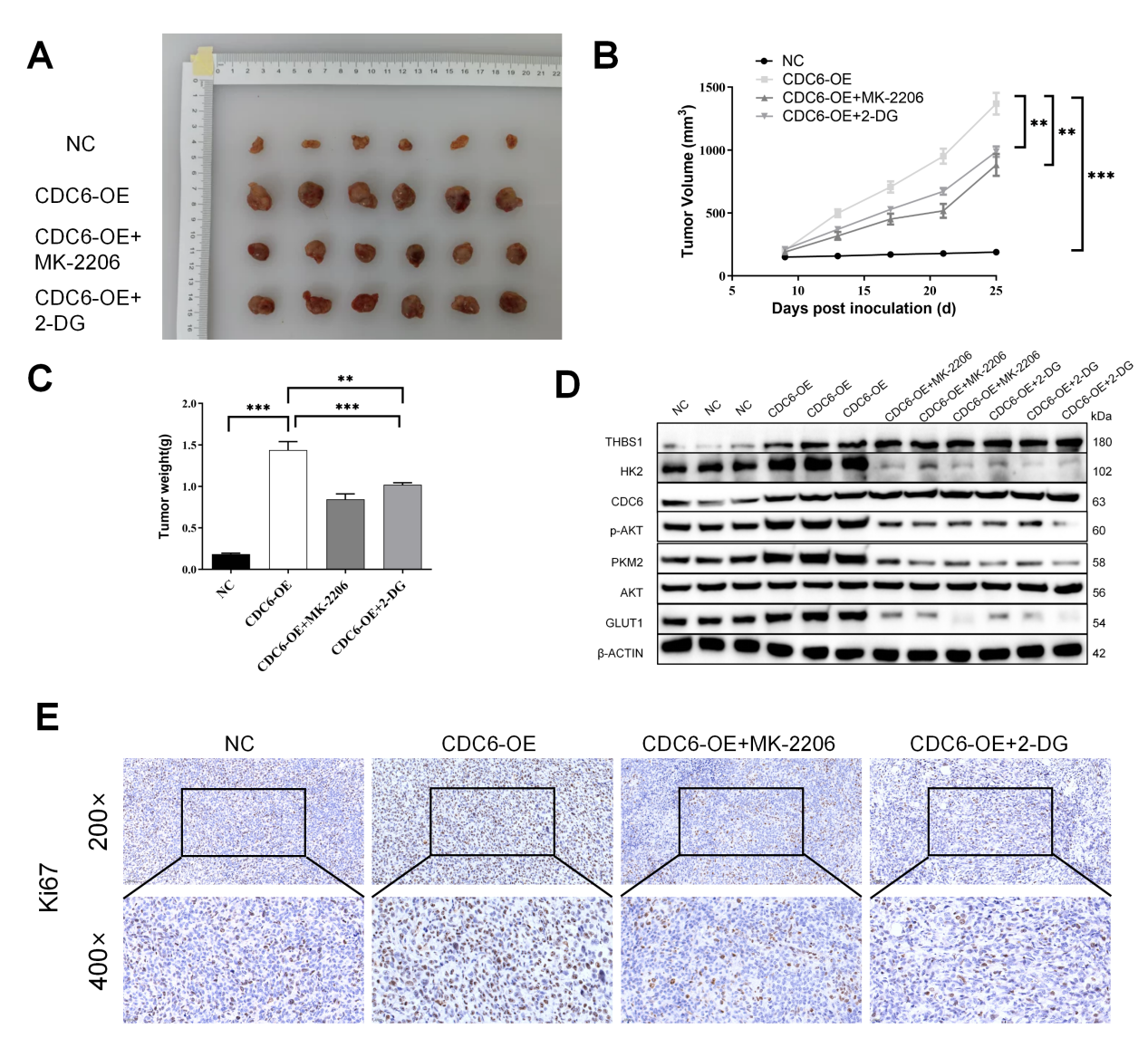
**

**Figure S5. CDC6 modulates glycolysis and malignant progression in vivo via AKT signaling pathway in pancreatic cancer. A.** 1×10^7^ NC or CDC6-OE or CDC6-OE+MK-2206 or CDC6-OE+2-DG PATU8988 cells were injected into the armpit of 6-week-old female Nude mice to induce xenograft tumor. Tumor images from four experimental groups (n=6) at the end of the experiment are shown. **B.**Tumor growth curves were made by the tumor volume measured between the 25 days after injection**. C.**Mice were sacrificed at day 25 post injection, and the final tumor weight was measured in each group. **D.**Western blot analysis of glycolytic enzymes and p-AKT levels in each group. **E.**IHC staining was performed in mouse tumor tissues to clarify the expressions of KI67. **Data expressed as mean ± SD. *P<0.05, **P<0.01, ***P<0.001.**
