# Supplementary material for: CDC6/THBS1 accelerates pancreatic cancer progression via AKT-mediated glycolytic reprogramming
Source: Cell Death Dis. 2026 Apr 21;17(1):524. doi: 10.1038/s41419-026-08758-2 (PMC13230735; doi:10.1038/s41419-026-08758-2)
Supplement: Supplementary file 3 — Supplementary Tables [file 41419_2026_8758_MOESM3_ESM.docx]

**Table 1S. PCR Primers**

| **Gene** | **Upstream primer sequence** | **Downstream primer sequence** |
| --- | --- | --- |
| GAPDH | 5’-TGACTTCAACAGCGACACCCA-3’ | 5’-CACCCTGTTGCTGTAGCCAAA-3’ |
| CDC6 | 5’-CTACACTCGGAACAAGGCAATGA-3’ | 5’-CTTCTGGCTGGTGATGGCTTC-3’ |
| THBS1 | 5’-CGTCCCATCCCGAAGAGATGAA-3’ | 5’-ATGAGTGCCGCTGCCTAGTG-3’ |

**Table 2S. WB Antibody Information**

| **Antibody target** | **Dilution ratio** | **Company** | **Item number** |
| --- | --- | --- | --- |
| CDC6 | 1:500 | Proteintech | 11640-1-AP |
| PKM2 | 1:5000 | Proteintech | 15822-1-Ig |
| HK2 | 1:3000 | Proteintech | 66974-1-Ig |
| GLUT1 | 1:1000 | Proteintech | 21829-1-AP |
| G6PD | 1:1000 | Abcam | ab210702 |
| FBP1 | 1:2000 | Proteintech | 12842-1-AP |
| E2F1 | 1:2000 | CST | 3742 |
| THBS1 | 1:500 | CST | 37879 |
| THBS1 | 1:1000 | BOSTER | BA2130-2 |
| AKT | 1:3000 | Proteintech | 10176-2-AP |
| p-AKT | 1:1000 | Proteintech | 66444-1-Ig |
| Histone H3 | 1:2000 | CST | 4499S |
| GAPDH | 1:30000 | Proteintech | 60004-1-lg |
| β-Actin | 1:2000 | Proteintech | 66009-1-Ig |
| Goat Anti-Rabbit | 1:3000 | Beyotime | A0208 |
| Goat Anti-Mouse | 1:3000 | Beyotime | A0216 |

**Table 3S. Knockdown sequences used for plasmid construction**

| **Sequence function** | **Sequence** |
| --- | --- |
| shCDC6 | 5’-CTGGACAATGCTGCAGTTCAA-3’，  5’-GAGGGTTGGTCTTATTCACAT-3’ |
| shTHBS1 | 5’-GGCCAACAAACAGGTGTGCAA-3’ |

**Table 4S. ChIP Antibody Information**

| **Antibody information** | **Antibody catalog number** | **Production company** |
| --- | --- | --- |
| Normal Rabbit IgG | 2729 | CST |
| Anti-E2F1 Antibody | BM4848 | BOSTER |
