# Supplementary material for: CDC6/THBS1 accelerates pancreatic cancer progression via AKT-mediated glycolytic reprogramming
Source: Cell Death Dis. 2026 Apr 21;17(1):524. doi: 10.1038/s41419-026-08758-2 (PMC13230735; doi:10.1038/s41419-026-08758-2)
Supplement: Supplementary file 4 — Supplementary material for WB [file 41419_2026_8758_MOESM4_ESM.docx]

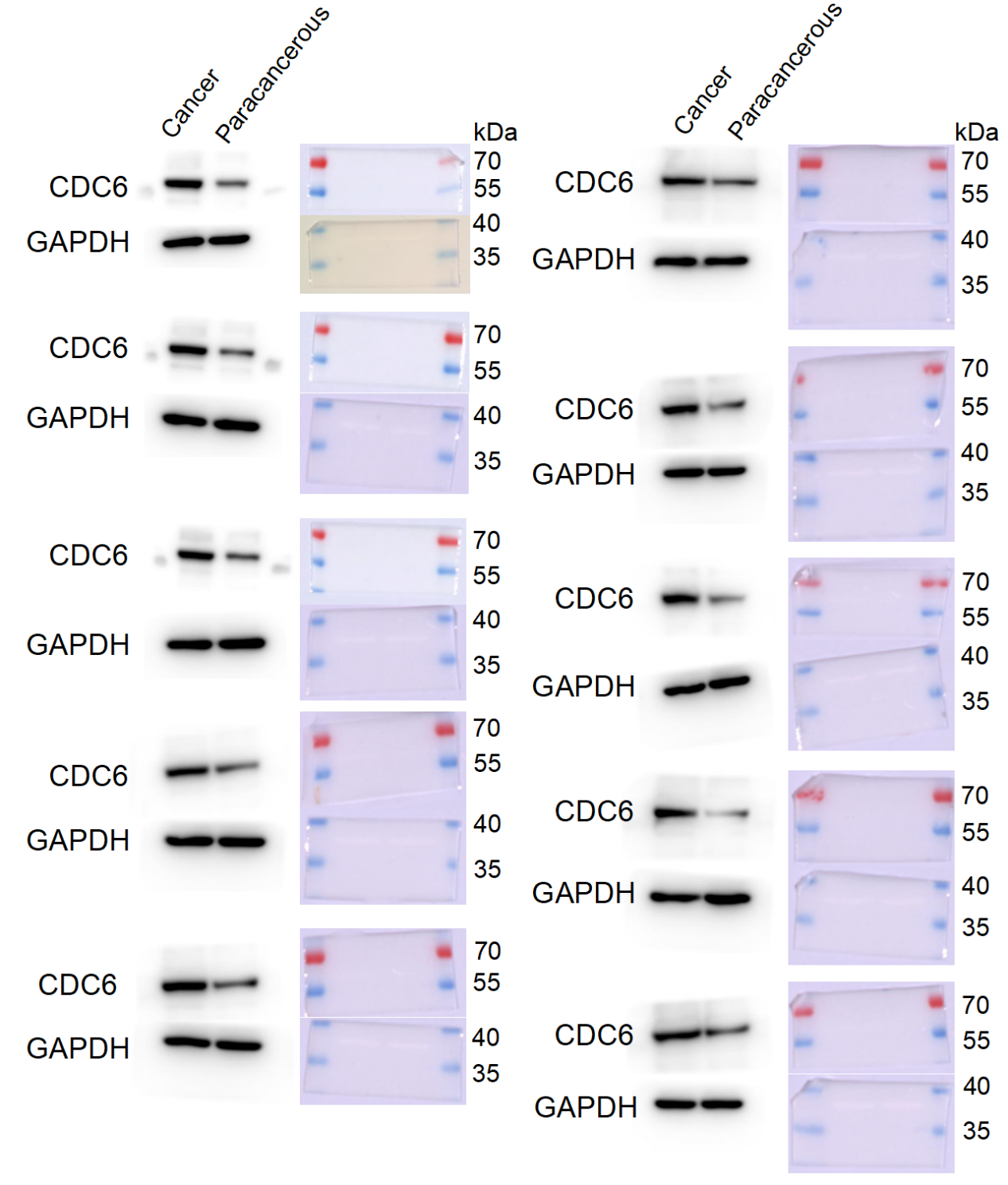


**Figure WB1: The original data of the result of WB in Figure 1D.**


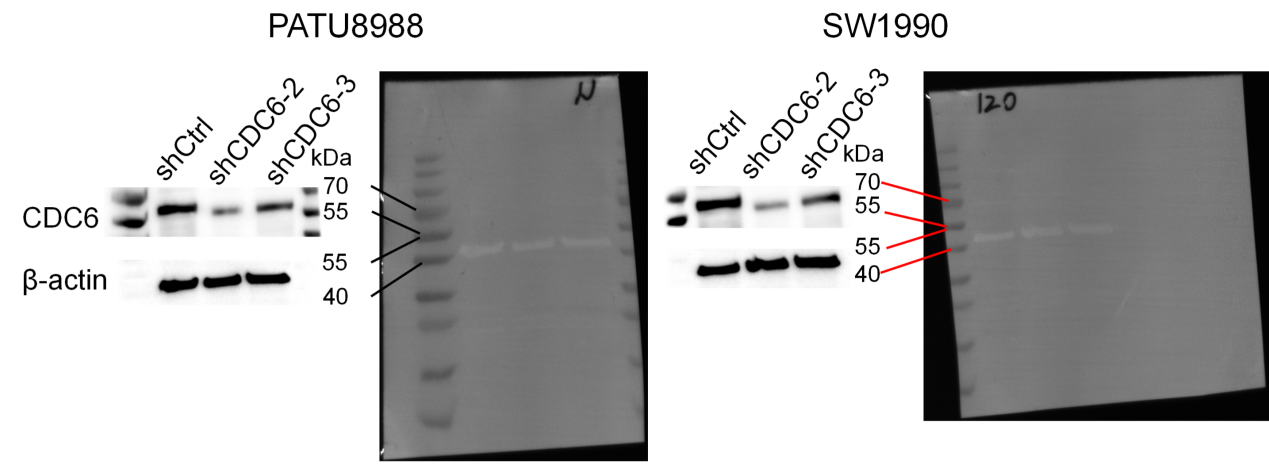


**Figure WB2: The original data of the result of WB in Figure 2B.**


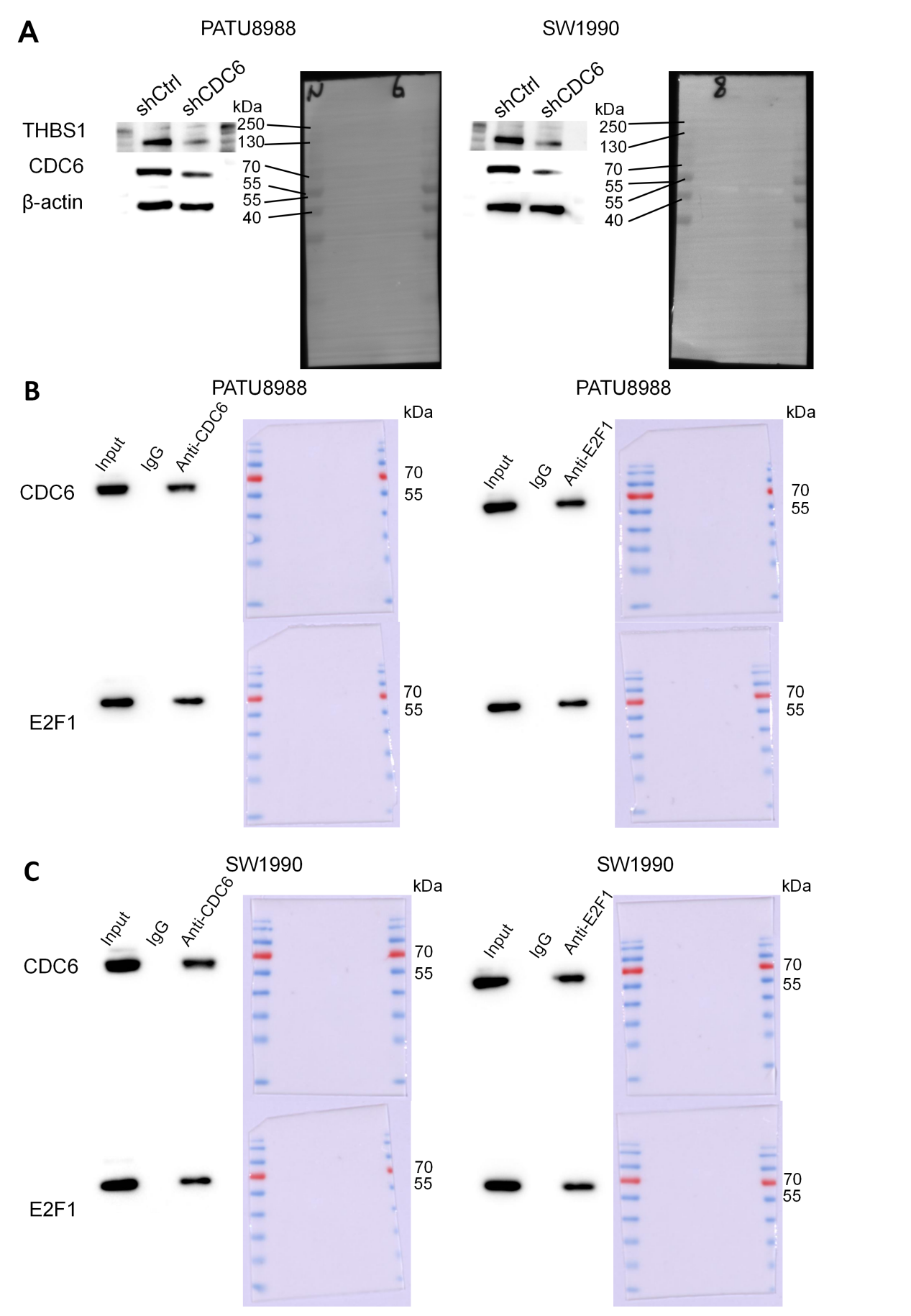


**Figure WB3: The original data of the result of WB in Figure 3B, D, E.** **A.** The original data of the Figure 3B. **B.** The original data of the Figure 3D. **C.** The original data of the Figure 3E.


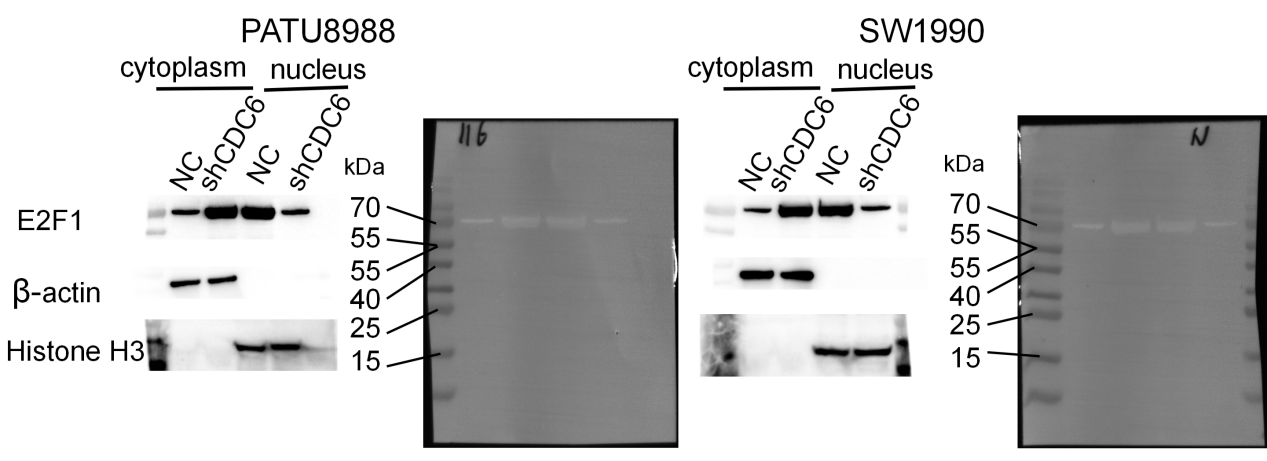


**Figure WB4: The original data of the result of WB in Figure 3H.**

**
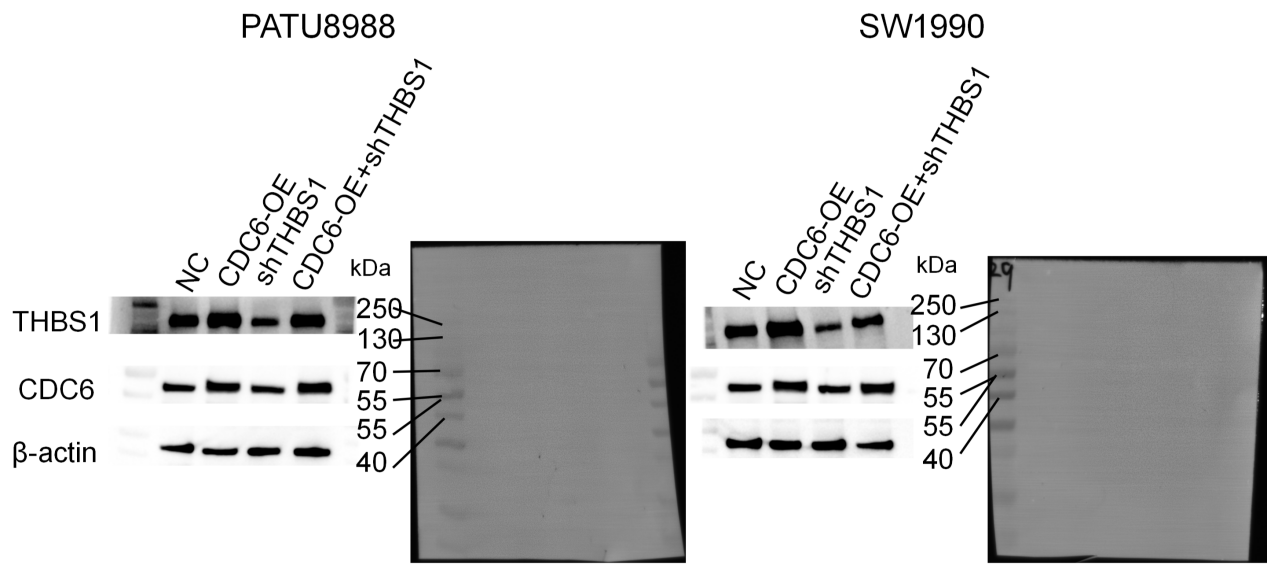
**

**Figure WB5: The original data of the result of WB in Figure S2 E-F.**

**
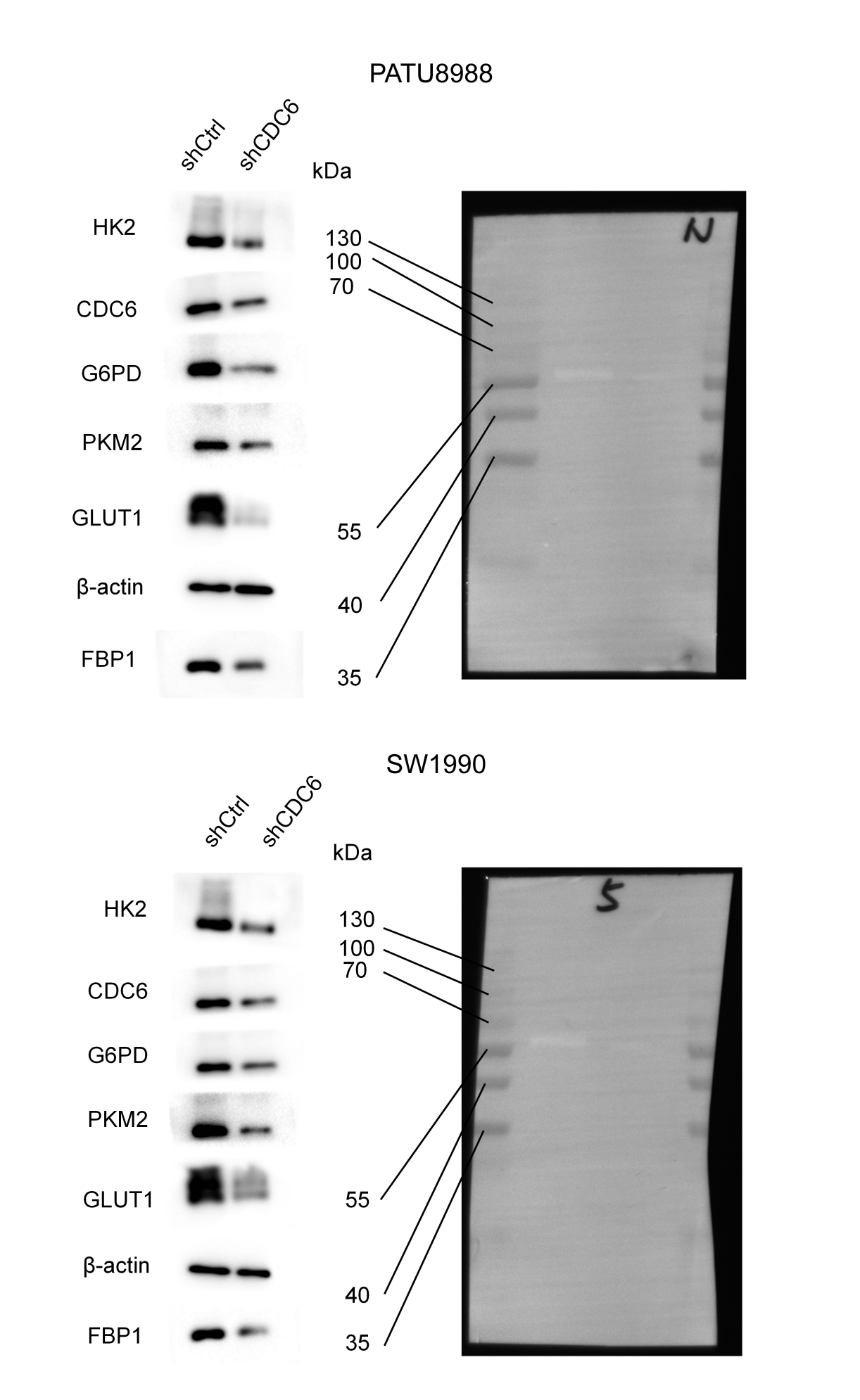
**

**Figure WB6: The original data of the result of WB in Figure 4A.**


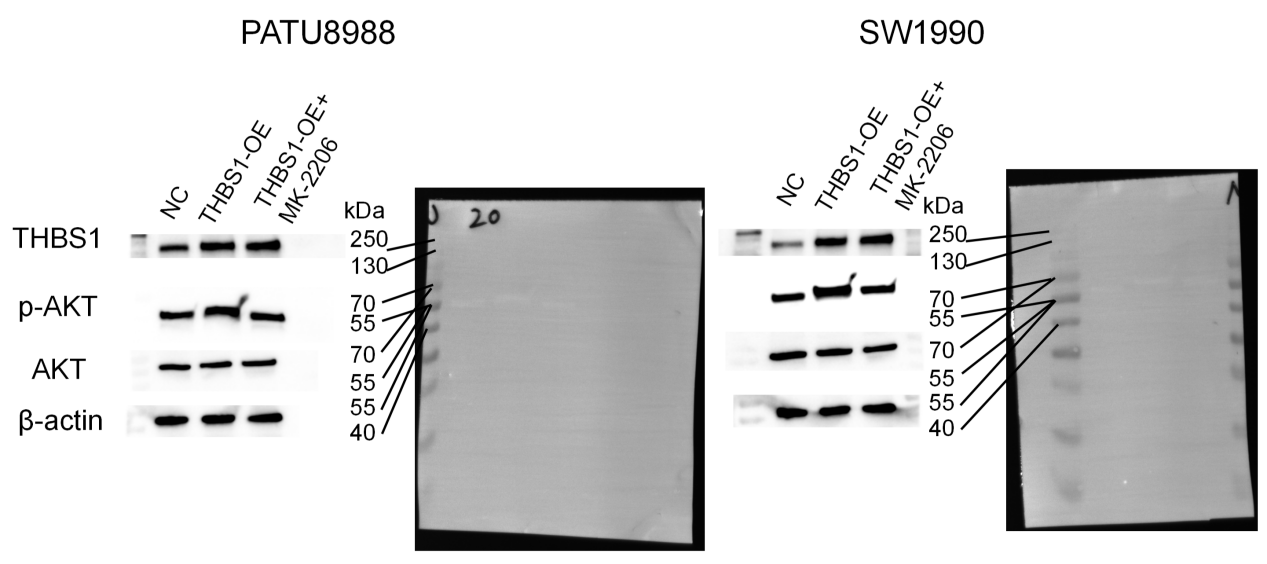


**Figure WB7: The original data of the result of WB in Figure 5C.**


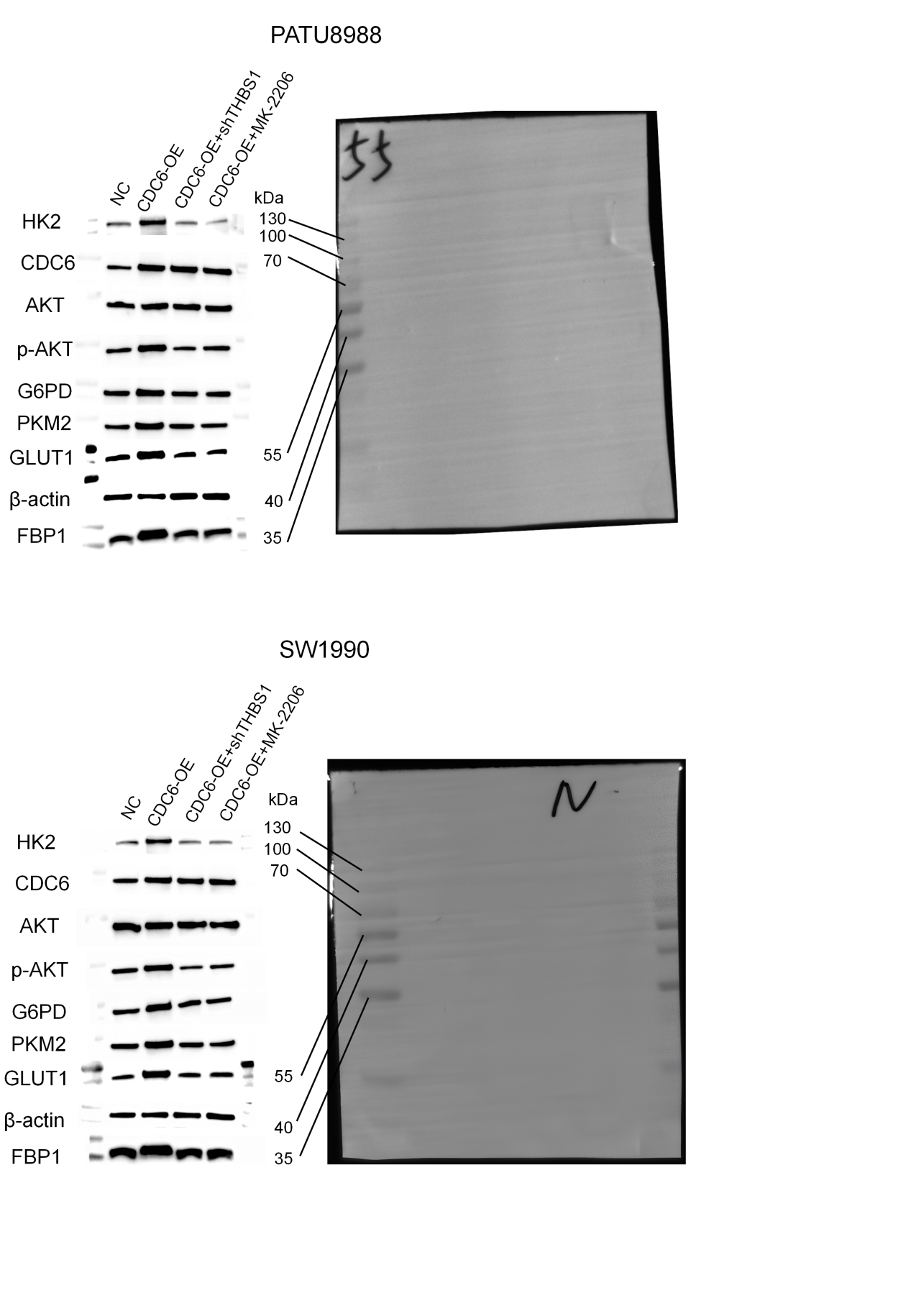


**Figure WB8: The original data of the result of WB in Figure 5D.**


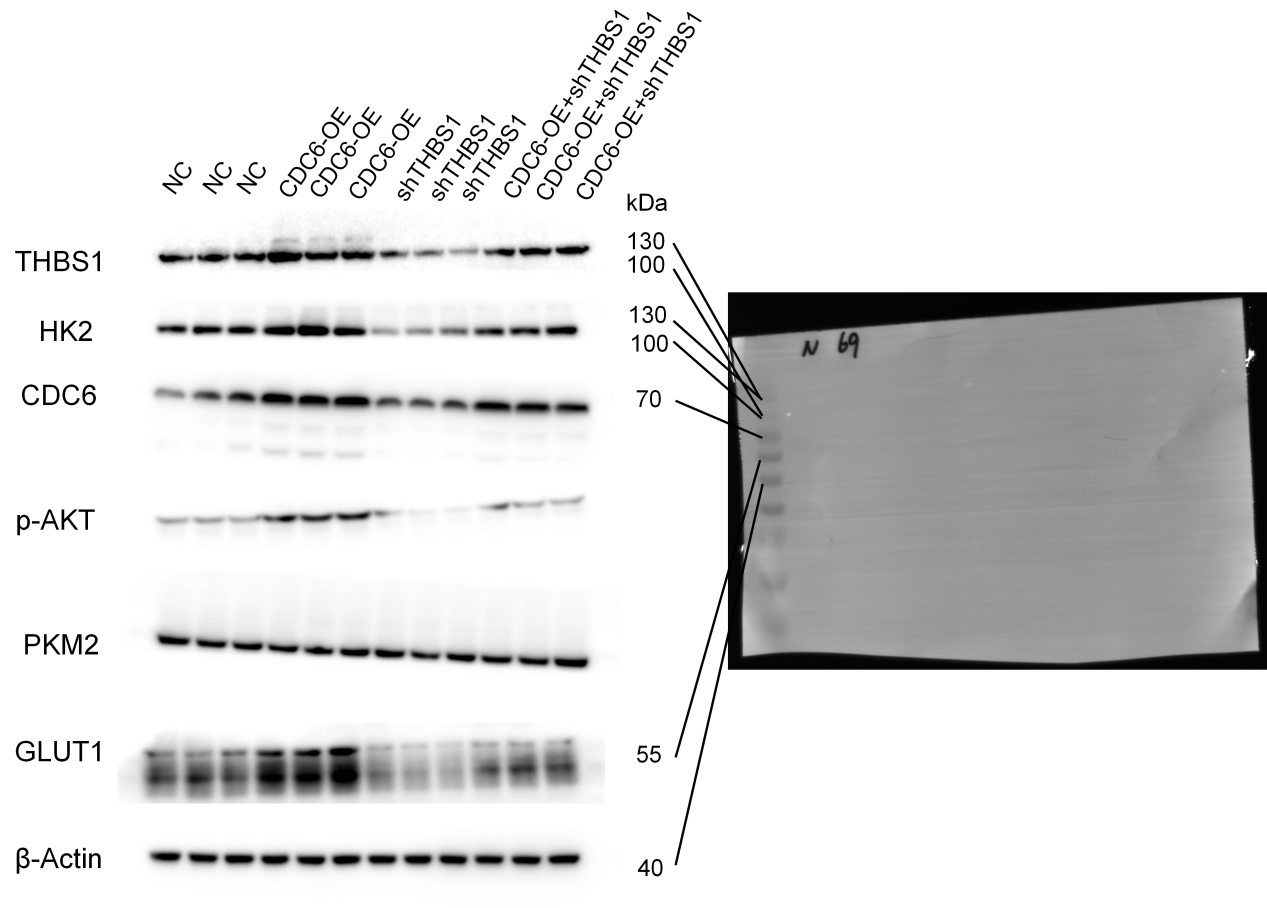


**Figure WB9: The original data of the result of WB in Figure 6D.**

**
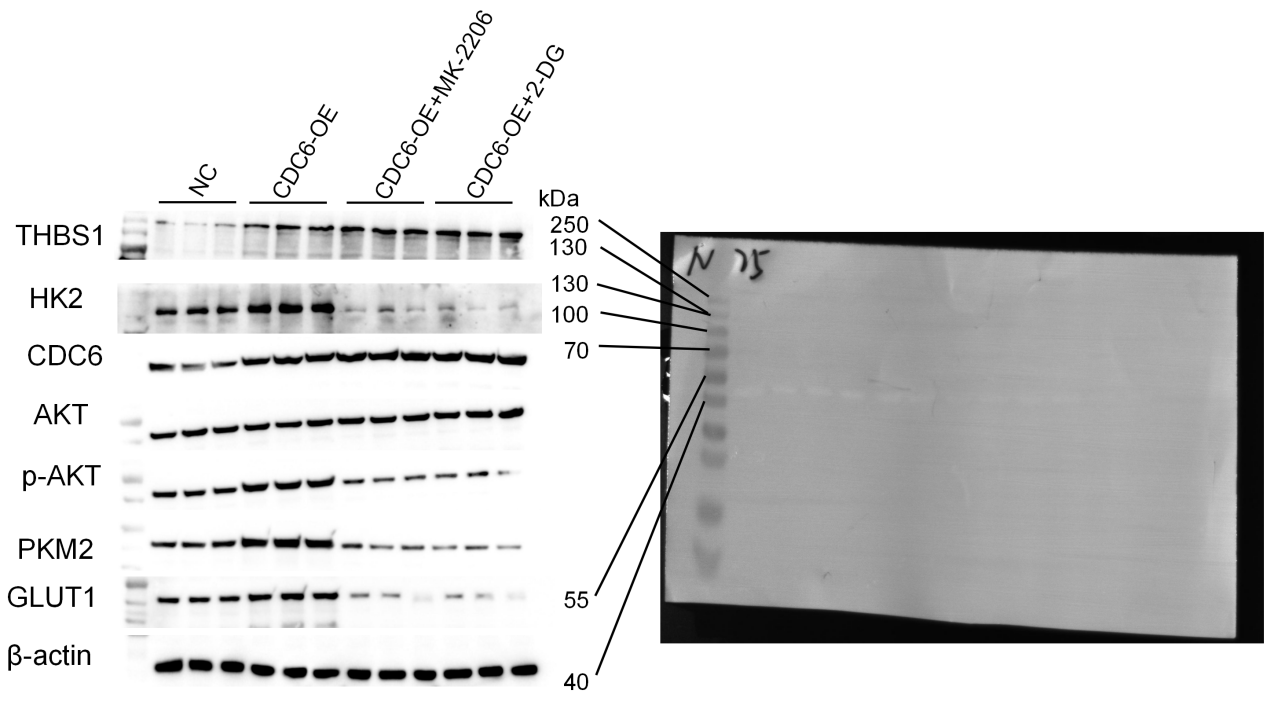
**

**Figure WB10: The original data of the result of WB in Figure S5D.**
